# Bleiverbindungen, organische: MAK-Begründung

**DOI:** 10.34865/mb7800d9_4or

**Published:** 2024-12-23

**Authors:** Andrea Hartwig, MAK Commission

**Affiliations:** 1 Institut für Angewandte Biowissenschaften. Abteilung Lebensmittelchemie und Toxikologie. Karlsruher Institut für Technologie (KIT) Adenauerring 20a, Geb. 50.41 76131 Karlsruhe Deutschland; 2 Ständige Senatskommission zur Prüfung gesundheitsschädlicher Arbeitsstoffe. Deutsche Forschungsgemeinschaft, Kennedyallee 40, 53175 Bonn, Deutschland. Weitere Informationen: Ständige Senatskommission zur Prüfung gesundheitsschädlicher Arbeitsstoffe | DFG

**Keywords:** Bleiverbindungen, organische, Bleitetramethyl, Bleitetraethyl, Neurotoxizität, zentrales Nervensystem, MAK-Wert, maximale Arbeitsplatzkonzentration, Kanzerogenität, Keimzellmutageniät, Entwicklungsneurotoxizität, Luft, Luft, Luft, air, air, air

## Abstract

The German Senate Commission for the Investigation of Health Hazards of Chemical Compounds in the Work Area (MAK Commission) summarized and re-evaluated the data for organic lead compounds [e.g. 78-00-2, 75-74-1, 17393-68-9] to derive an occupational exposure limit value (maximum concentration at the workplace, MAK value) considering all toxicological end points. Relevant studies were identified from a literature search. The analyses of urine and faeces after exposure to tetraethyllead or tetramethyllead showed metabolism to inorganic lead. The significantly higher lipophilicity of tetraethyllead and tetramethyllead results in a higher uptake into the brain compared to lead ions and suggests a better uptake in neurons. This might explain the stronger neurotoxic effects observed after exposure to organic lead compounds. Reliable data to derive a MAK value for tetraethyllead, tetramethyllead and other organic lead compounds are not available. As metabolism leads to the formation of inorganic lead, at least the MAK value for inorganic lead must be met and therefore this value is adopted and the MAK value of tetraethyllead and tetramethyllead is lowered to 0.004 mg/m^3^, as lead. This MAK value also applies to other organic lead compounds. In analogy to the classifications made for inorganic lead compounds, the substances have been assigned to Carcinogen Category 4 and Germ Cell Mutagenicity Category 3 A. As it has been demonstrated unequivocally that lead induces damage to the human embryo and foetus and that these effects are to be expected even if the MAK and biological tolerance (BAT) values are not exceeded, lead has been assigned to Pregnancy Risk Group A. In analogy to inorganic lead, organic lead compounds have likewise been assigned to Pregnancy Risk Group A. According to skin absorption models, percutaneous absorption is expected to contribute significantly to systemic toxicity. Therefore, organic lead compounds have been designated with “H”. There are no data for respiratory or skin sensitization.

**Table:** 

**MAK-Wert (2023)**	**0,004 mg/m^3^ (als Pb berechnet)**
**Spitzenbegrenzung (2001)**	**Kategorie II, Überschreitungsfaktor 1**
	
**Hautresorption (1966)**	**H**
**Sensibilisierende Wirkung**	**–**
**Krebserzeugende Wirkung (2023)**	**Kategorie 4**
**Fruchtschädigende Wirkung (2023)**	**Gruppe A**
**Keimzellmutagene Wirkung (2023)**	**Kategorie 3 A**
	
**BLW (2024)**	**Bleitetraethyl/Bleitetramethyl: 150 µg Gesamtblei/l Urin**
	
**1 ml Bleitetraethyl/m^3^ (ppm) ≙ 13,42 mg/m^3^**	**1 mg Bleitetraethyl/m^3^ ≙ 0,0745 ml/m^3^ (ppm)**
**1 ml Bleitetramethyl/m^3^ (ppm) ≙ 11,09 mg/m^3^**	**1 mg Bleitetramethyl/m^3^ ≙ 0,0902 ml/m^3^ (ppm)**

**Tab. 1:** Organische Bleiverbindungen mit Daten zur toxischen Wirkung

**Stoff** **Verwendung**	**CAS-Nr.**	**IUPAC-Name**	**Formel**	**Dampfdruck**	**Log K_OW_**	**Löslichkeit**
Bleitetraethyl Benzinzusatz	78-00-2	Tetraethylplumban	Pb(C_2_H_5_)_4_ C_8_H_20_Pb	0,3466 hPa (bei 25 °C, ATSDR 2020)	4,15 (ATSDR 2020)	0,29 mg/l Wasser (ATSDR 2020)
Bleitetramethyl Benzinzusatz	75-74-1	Tetramethylplumban	Pb(CH_3_)_4 _ C_4_H_12_Pb	32 hPa (bei 20 °C, IFA 2023 b)	2,97 (IFA 2023 b)	300 mg/l Wasser (IFA 2023 b)
Triethylneopentoxyblei Herstellung von Dünnschichtfilmen	17393-68-9	(2,2-Dimethylpropoxy)(triethyl)plumban	(C_2_H_5_)_3_Pb[OCH_2_C(CH_3_)_3_] C_11_H_26_OPb	k. A.	k. A.	k. A.

Hinweis: In Abhängigkeit vom Dampfdruck können manche Verbindungen gleichzeitig als Dampf und Aerosol vorliegen.

Zu Bleitetraethyl liegen eine Begründung (Henschler 1972 a), ein Nachtrag (Greim 1994), ein Nachtrag zur Spitzenbegrenzung (Greim 2001 a) und eine Bewertung der Entwicklungstoxizität (Hartwig 2009 a) vor.

Zu Bleitetramethyl liegen eine Begründung (Henschler 1972 b), ein Nachtrag (Greim 1995), ein Nachtrag zur Spitzenbegrenzung (Greim 2001 b) und eine Bewertung der Entwicklungstoxizität (Hartwig 2009 b) vor.

Die meisten Daten liegen zu Bleitetraethyl und Bleitetramethyl vor.

**Bleiacetat** ist eine ionische Verbindung, die daher wie die anorganischen Bleiverbindungen zu bewerten ist (Hartwig und MAK Commission 2022).

Zur Herstellung dünner ferroelektrischer Schichten durch Aufdampfen werden Bleitetraethyl und weitere organische Bleiverbindungen in der Chip-Industrie verwendet (Arai et al. 1998).

Nach den 1920er Jahren wurde die Umweltverschmutzung durch Blei, die durch die Einführung von Bleitetraethyl als Benzinzusatzstoff verursacht wurde, zu einem Problem für die öffentliche Gesundheit. Die Verwendung wurde in den 1980er Jahren stark eingeschränkt und in der Folge sank der Bleigehalt des Blutes in der Bevölkerung deutlich (Hernberg 2000).

Produktion und Vertrieb von verbleitem Benzin wurden in Deutschland 1996 freiwillig eingestellt. Seit dem 1. Januar 2000 ist das Inverkehrbringen von verbleitem Ottokraftstoff gesetzlich verboten (BUA 2000). Seit 2021 ist bleihaltiges Benzin weltweit verboten. Bleitetraethyl steht seit April 2022 auf der Zulassungsliste der EU (DNR 2022; ECHA 2022 a).

Ein Großteil des in Benzin enthaltenen Bleitetraethyls/-methyls wird durch den Verbrennungsvorgang zu anorganischen Bleiverbindungen umgewandelt. Durch unvollständige Verbrennung und Verdunstung gelangten Bleitetraethyl und gegebenenfalls Bleitetramethyl in die Luft (Nielsen et al. 1978).

Bleitetraethyl und -methyl werden weiterhin von zwei Firmen in Frankreich und einer polnischen Firma hergestellt und als Benzinzusatz in AvGas („aviation gasoline“) bei Leichtflugzeugen mit Kolbenmotoren eingesetzt. Es ist ebenfalls als Laborreagenz erhältlich (DNR 2022; ECHA 2022 b).

Da neue Studien zu organischen Bleiverbindungen vorliegen und sich für Blei und seine anorganischen Verbindungen der BAT- und der MAK-Wert, die Kanzerogenitäts-Kategorie und die Schwangerschaftsgruppe geändert haben, wird eine Neubewertung vorgenommen.

## 1 Allgemeiner Wirkungscharakter

Bleitetraethyl und Bleitetramethyl wirken neurotoxisch auf das zentrale (ZNS) und periphere Nervensystem (PNS) bei Mensch und Tier. Symptome einer schweren akuten Vergiftung sind akustische und optische Halluzinationen, Krampfanfälle, motorische Störungen und Sprachstörungen sowie Delirium und Koma. Langjährige Exposition gegen 0,05–0,069 mg/m^3^ haben bei Beschäftigten eines Benzindepots zu einer statistisch signifikant erhöhten Tremor-Häufigkeit geführt.

Der Metabolismus erfolgt über Dealkylierung. Bei Ratten treten nach 13-wöchiger oraler Gabe von Trialkylblei bei einer Dosis von 0,05 mg/kg KG und Tag schon Rückenmarksläsionen auf. Zudem reichert sich Trialkylblei im Vergleich zu Blei aus Bleisalzen deutlich stärker im Gehirn an.

Bleitetraethyl und Bleitetramethyl werden aufgrund ihrer lipophilen Eigenschaften gut über die Haut aufgenommen.

Die beim metabolischen Abbau von Bleitetraethyl und Bleitetramethyl entstehenden anorganischen Blei-Ionen wirken kanzerogen und mutagen in Keimzellen. 

Es liegen keine Studien mit organischen Bleiverbindungen zur Entwicklungsneurotoxizität vor. Aufgrund der Entstehung anorganischer Blei-Ionen beim metabolischen Abbau von Bleitetraethyl und Bleitetramethyl ist von einer entwicklungsneurotoxischen Wirkung auszugehen.

Zur sensibilisierenden Wirkung liegen weder Daten noch Verdachtsmomente vor.

## 2 Wirkungsmechanismus

Aus zahlreichen Vergiftungsfällen sind die neurotoxischen Wirkungen des Bleitetraethyls und -methyls auf das ZNS und PNS bekannt. Ausführliche Darstellungen finden sich in Bolt (1996) und Greim (1994).

Da Ratten Symptome wie unkontrollierte aggressive Aktivität, Desorientierung und Krämpfe nach einmaliger oraler Gabe von Bleitetraethyl zeigten, die auch nach schweren Bleitetraethyl-Vergiftungen beim Menschen beschrieben sind (Haskell Laboratories 1992 b), können Versuche zur Neurotoxizität an Ratten zur Aufklärung des Wirkungsmechanismus beitragen.

Bleitetraethyl führte an isolierten Lungenmitochondrien zu einer beeinträchtigten mitochondrialen Funktion, was auf den Verlust der oxidativen Phosphorylierungskapazität zurückgeführt wird (k. w. A.; Simões et al. 2010). Die Publikation liegt nur als Abstract vor.

Bleitetraethyl steht im Verdacht epigenetische Veränderungen hervorzurufen. Durch Missbrauch von Bleitetraethyl-enthaltendem Benzin (Schnüffeln) wurde bei 19 Personen ein mittlerer Blutbleiwert von 481 µg/l ermittelt und in einer weiteren Untersuchung bei 25 Personen ein Blutbleiwert > 500 µg/l. Die Autoren vermuten, dass Bleitetraethyl nach der Metabolisierung zu einer Überexpression des Amyloid-β-Proteinvorläufers (AβPP) und des Amyloid-β (Aβ) beiträgt, was zu einer verstärkten pathologischen Neurodegeneration führen kann (Fonseka et al. 2017).

Neurotoxisch wirksam ist bei den Bleitetraalkyl-Verbindungen der Metabolit Trialkylblei, der sich aufgrund seiner ionischen Ladung schneller im Körper verteilt als Bleitetraalkyl (Bolanowska 1968; Henschler 1972 a). Daher werden im Folgenden auch Studien mit Trialkylblei dargestellt.

Bleitrialkyle (Bleibutyl, -ethyl- und -methyl) induzierten bei aus Rattenleber isolierten Mitochondrien ein Anschwellen durch Öffnen der Transmembran-Pore, das zu einer Hemmung der ATP-Synthese führt. Der dadurch veränderte Energiehaushalt wird für den Zelltod verantwortlich gemacht (Bragadin et al. 2007).

Histopathologische Untersuchungen sieben und 28 Tage nach subkutaner Injektion von Trialkylblei-Verbindungen an männliche Ratten zeigten, dass Trimethylblei (8,8 bis 26,2 mg/kg KG) in erster Linie Veränderungen am Rückenmark und im Hirnstamm verursachte, während Triethylblei (2,6–7,9 mg/kg KG) strukturelle Änderungen des Hippocampus und der Hinterwurzelganglien hervorrief (Greim 1995).

Trialkylbleichlorid wurde in In-vitro-Versuchen mit Rattenleber-Mitochondrien als (Alkyl)_3_Pb^+^-Kation aufgenommen, jedoch als neutrales (Alkyl)_3_Pb-OH wieder ausgeschleust. Es wird vermutet, dass die Aufnahme von Trialkylblei-Verbindungen in Zellen durch ein negatives Potential verstärkt wird. Dies könnte ihre bevorzugte Toxizität für neuronale Zellen erklären, die ein höheres negatives Potential im Zellinneren haben als viele andere Zelltypen (Bragadin et al. 1998).

Kultivierte E18-Rattenhippocampus-Neuronen zeigten nach Inkubation mit 10 nM Triethylblei eine inhibierte Dendritenverzweigung und mit 100 nM Triethylblei eine inhibierte Axon-Verzweigung. Eine reduzierte Länge der Axone wurde ab 1 µM Triethylblei und reduziertes Wachstum der Neuronen ab 5 µM Triethylblei beobachtet (Audesirk et al. 1995).

Trialkylblei führte nach oraler Gabe bei jungen Ratten zu einer Abnahme an Neurofilamenten. Die Autoren gehen davon aus, dass die Bildung von Neurofilamenten durch Trialkylblei gestört wird und damit auch der axonale Transport (Yagminas et al. 1992).

Der neurotoxische Effekt von Trimethylblei wurde in In-vitro-Versuchen auf die Reduzierung der Ströme durch den spannungsabhängigen Calciumkanal von kultivierten Spinalganglion-Neuronen zurückgeführt (Gawrisch et al. 1997).

Triethylblei hemmte die membrangebundene Na^+^-K^+^-ATPase in HeLa-Zellen (IC_50_ 12 µM) und die ATP-hydrolysierende Aktivität des mitochondrialen F0-F1-ATPase-Komplexes (IC_50_ 17 µM) (Münter et al. 1989).

Triethylblei rief in HL-60-Zellen einen anhaltenden Anstieg der zytosolischen freien Calciumkonzentration und eine Zunahme an freier Arachidonsäure hervor. Die beobachtete Freisetzung von Arachidonsäure durch eine Aktivierung von Phospholipase A2 war auf eine erhöhte intrazelluläre Ca^2+^-Konzentration durch einen Ca^2+^-Einstrom aus dem extrazellulären Raum zurückzuführen. Basierend auf der wesentlichen Rolle von Ca^2+^ innerhalb der zellulären Signalübertragung scheint die Störung der Calciumhomöostase ein wichtiges Ereignis bei der endgültigen Zelltötung durch Organometalle zu sein. Andere biochemische Mechanismen, wie die Aktivierung der Phospholipase A2, werden möglicherweise durch einen Anstieg von Ca^2+^ vermittelt (Ade et al. 1996; Käfer und Krug 1994).

Die stärkeren lipophilen Eigenschaften von Bleitetraethyl und Bleitetramethyl im Vergleich zu Blei-Ionen führen zu einer höheren Wirkstärke. Dies zeigen die im Folgenden dargestellten Versuche.

An präparierten Gehirnschnitten des Cortex von männlichen Wistar-Ratten hemmte die Inkubation mit 1–100 mM Triethylbleiacetat konzentrationsabhängig die Freisetzung und Synthese von Acetylcholin. Isolierte Cholinacetyltransferase wurde durch 10^–5^ M Triethylblei gehemmt, jedoch durch die ionische Bleiverbindung **Bleiacetat** erst bei höheren Konzentrationen von 10^–4^ M (Hoshi et al. 1991).

Tributylblei hemmte bereits ab 10^–8^ M die Dopaminaufnahme und Freisetzung in Gehirnhomogenaten von Mäusen (A/J-Stamm), Bleiacetat erst ab Konzentrationen, die höher als 10^–6^ M lagen. Die Neurotoxizität von organischen Bleiverbindungen ist nach Meinung der Autoren eher ein akuter Effekt, dem eine massive Zellschädigung zugrunde liegt. Damit kann nach Meinung der Autoren diese Wirkung dem neurotoxischen Mechanismus eines Excitotoxins zugeordnet werden (Bondy et al. 1979; Verity 1990). Ein Excitotoxin ist eine Substanz, welche durch Erregung von Nervenzellen deren Untergang verursacht.

Nach intraperitonealer (i.p.) Gabe von 42,7 mg Bleitetraethyl/kg KG in Maiskeimöl an 40 Meerschweinchen erfolgte eine Untersuchung der Cochlea nach 120 Minuten. Bei gleicher Blei-Konzentration verursachte Bleitetraethyl eine stärkere Dysfunktion der Cochlea als **Bleiacetat** (Tuncel et al. 2002).

**Fazit**: Orale Tierstudien zeigen eine höhere Empfindlichkeit neuronaler Zellen gegenüber Bleitetraethyl und Triethylblei im Vergleich mit Bleiacetat bzw. Blei-Ionen. Die Änderung bzw. Hemmung neuronaler Funktionen durch Blei-Ionen werden auf zahlreiche Mechanismen zurückgeführt (Greiner et al. 2022). Durch die hohe Lipophilie und die damit verbundene schnelle Aufnahme hoher Konzentrationen Bleitetraethyls oder Bleitetramethyls in neuronale Zellen kommt es hier zu stärkeren Blei-induzierten Veränderungen. Anzunehmen ist auch, dass eine massive Zellschädigung auftritt, die bei Exposition gegen Blei-Ionen nicht beobachtet wurde. Wie bei anorganischen Bleiverbindungen (Greiner et al. 2022) wirken auch Bleialkylverbindungen auf die intrazelluläre Calciumhomöostase und beeinträchtigen mitochondriale Membranpotentiale und den Energiehaushalt der Zelle.

Bleitetraethyl und Bleitetramethyl zeigen verschiedene Wirkungen und Wirkorte in Bezug auf die Neurotoxizität. Dies wird durch unterschiedliche Effekte der jeweiligen Trialkyle bestätigt.

## 3 Toxikokinetik und Metabolismus

### 3.1 Aufnahme, Verteilung, Ausscheidung

#### 3.1.1 Mensch

Bleitetraethyl und Bleitetramethyl können als Dampf vorliegen und dadurch unterscheiden sie sich in der Resorption von eingeatmeten anorganischen Bleipartikeln (IARC 2006). Für inhalierte partikuläre anorganische Bleiverbindungen wird eine Resorption (und Retention) von 30 % angenommen (Hartwig und MAK Commission 2022).

Bei inhalativer Exposition werden etwa 80 % des Bleitetraethyls resorbiert (Schätzung unter der Annahme, dass alles Bleitetraethyl mit der alveolären Ventilation aufgenommen wird) (k. w. A.; Grandjean und Nielsen 1979; Greim 1994).

Vier männliche Freiwillige wurden gegen 1 mg ^203^Pb-Bleitetraethyl/m^3^ oder ^203^Pb-Bleitetramethyl/m^3^ 1–2 Minuten bzw. 10–40 Atemzüge lang exponiert. Es wurden 37 % Bleitetraethyl resorbiert, davon wurden 20 % innerhalb von 48 Stunden wieder abgeatmet. Eine Stunde nach der Exposition waren etwa 50 % der ^203^Pb-Belastung mit der Leber und 5 % mit der Niere assoziiert. Die verbleibende Belastung war über den Körper verteilt. Die Bestimmung wurde mittels externer Radioaktivitätsmessung vorgenommen. Die Zerfalls-Halbwertszeit von ^203^Pb beträgt 52 Stunden. Nach zwölf Tagen war keine Radioaktivität mehr messbar. Die Inhalation von 1 mg ^203^Pb-Bleitetramethyl/m^3^ führte zu einer 51%igen Resorption, davon wurden innerhalb von 48 Stunden 40 % wieder abgeatmet. Im Blut der Probanden zeigte sich anhand der Kinetik von ^203^Pb nach der Exposition eine Phase abnehmender Blutbleikonzentration während der ersten vier Stunden (Bleitetramethyl) oder zehn Stunden (Bleitetraethyl). In dieser Phase wurde das Blei im Plasma detektiert. Danach folgte eine Phase mit allmählichem Anstieg der Blutbleikonzentration, die bis zu 500 Stunden andauerte. Das Blei befand sich in dieser Phase in den Erythrozyten. Radioaktives Blei im Blut war unmittelbar nach der Exposition hochflüchtig und ging danach in einen nichtflüchtigen Zustand über. Die Ausscheidung mit Urin und Faeces betrug täglich 0,2 %. Das Verhältnis der Ausscheidung im Stuhl/Urin betrug bei Bleitetraethyl 1:1,8 und bei Bleitetramethyl 1:1,0. Die Aufenthaltszeit („mean residence time“) im Blut wird mit 13 Sekunden angegeben (k. w. A.; ATSDR 2020; Greim 1994, 1995; Heard et al. 1979). Die Studie liegt nur als Konferenzbeitrag vor. Die Abnahme der Blutbleikonzentration in den ersten zehn Stunden kann mit der Aufnahme aus dem Plasma in die Leber erklärt werden. Die dort stattfindende Metabolisierung zu anorganischen Bleiverbindungen führte zu einer Aufnahme von Blei-Ionen in die Erythrozyten und damit zu einer wieder ansteigenden Blutbleikonzentration. Nach dieser Studie ist die inhalative Resorption von Bleitetraethyl und Bleitetramethyl 40 bzw. 50 % und damit höher als die von anorganischen Bleiverbindungen. Da jedoch beide resorbierten Bleialkyle mit 20 bzw. 40 % relativ schnell abgeatmet werden, ist der retinierte Prozentsatz bei Bleitetraethyl und Bleitetramethyl 30 % und damit so hoch wie die bei Hartwig und MAK Commission (2022) beschriebene Aufnahme für anorganische Bleiverbindungen.

Bleialkyle werden alten Angaben zufolge „unter praxisnahen Bedingungen“ zu ca. 6 % über die Haut aufgenommen (Greim 1994).

Berechnungen mit dem IH SkinPerm-Modell (Tibaldi et al. 2014) bzw. dem Modell nach Fiserova-Bergerova et al. (1990) unter Betrachtung gesättigter wässriger Lösungen ergeben dermale Fluxe von 1,7 bzw. 40 µg/cm^2^ und Stunde für Bleitetramethyl und 0,005 bzw. 0,16 µg/cm^2^ und Stunde für Bleitetraethyl. Dies würde Aufnahmemengen von 3,44 mg bzw. 79,3 mg Bleitetramethyl (2,67 bzw. 61,5 mg Blei) sowie 0,01 mg bzw. 0,3 mg Bleitetraethyl (0,0064 bzw. 0,19 mg Blei) nach einstündiger Exposition einer Hautfläche von 2000 cm^2^ bedeuten.

Autopsien des Gehirns von 22 Personen aus der Allgemeinbevölkerung (Kopenhagen, Dänemark) ergaben eine mediane Konzentration an **Trialkylblei** von 0,2 µmol pro kg (Nielsen et al. 1978).

Nach versehentlicher oraler oder inhalativer Aufnahme von Bleitetraethyl fanden sich bei zwei verstorbenen Patienten die höchsten Bleimengen in der Leber gefolgt von Niere, Gehirn, Pankreas, Muskeln und Herz (IARC 2006).

Am 21. Tag nach einer akuten Bleitetraethyl-Vergiftung wurden von der ausgeschiedenen Gesamtbleikonzentration von 382 µg/l 50 % als Diethylblei, 2 % als Triethylblei und 48 % als anorganisches Blei im Urin des Patienten bestimmt (Bolt 1996; Greim 1994; Yamamura et al. 1981).

In einer bereits bei Bolt (1996) und Greim (1994) geschilderten Untersuchung wurde die Korrelation zwischen Blei-Expositionskonzentrationen und der Ausscheidung von Diethylblei, Triethylblei und anorganischem Blei im Urin von Beschäftigten eines Benzindepots, Verkehrspolizisten und unbelasteten Büroangestellten in China untersucht. Die Daten sind in der Tabelle 2 dargestellt (Greim 1994; Zhang et al. 1994).

**Tab. 2:** Bleitetraethyl in der Luft (µg/m^3^ als Blei berechnet) und Bleitetraethylmetaboliten im Urin (Zhang et al. 1994)

**Untersuchungsgruppe**	**Anzahl Luftproben**	**Luftkonzentration Et_4_Pb** **[µg Pb/m^3^ ± SD]**	**Anzahl Urinproben**	**Urinkonzentration** **[µg Pb/l ± SD]**		
				**Et_2_Pb^2+^**	**Et_3_Pb^+^**	**Gesamtblei**
Kontrolle (Büroangestellte)	18	1,10 ± 0,4	342	3,82 ± 1,8	0,16 ± 0,10	16,9 ± 9,8
Verkehrspolizisten	12	5,20 ± 2,4	36	8,38 ± 5,6	0,26 ± 0,16	46,1 ± 16,6
Benzindepot-Beschäftigte	6	40,9 ± 9,9	27	18,8 ± 7,2	0,37 ± 0,17	58,2 ± 20,2
	8	61,1 ± 9,0	168	30,8 ± 12,3	0,46 ± 0,19	52,6 ± 22,3
	6	81,9 ± 8,8	51	42,9 ± 21,4	0,68 ± 0,24	71,8 ± 23,7
	16	106,9 ± 12,5	31	65,7 ± 11,9	0,83 ± 0,18	78,4 ± 20,8

Et_2_Pb^2+^: Diethylblei; Et_3_Pb^+^: Triethylblei; Et_4_Pb: Bleitetraethyl

Urinproben von 15 Tankstellen-Beschäftigten in Ankara (Türkei), die zehn Stunden/Tag beschäftigt waren und außerhalb der Stadt wohnten, wurden auf die Bleikonzentration untersucht. Die mittlere Gesamtblei-Konzentration betrug 79,0 (22,6–158,90) µg/g Kreatinin bzw. 111,9 (18,1–238,5) µg/l Urin und die Konzentration an anorganischem Blei 37,3 (5,1–121,0) µg/g Kreatinin bzw. 50,2 (4,1–180,0) µg/l Urin. Bei den Kontrollpersonen wurde im Urin anorganisches Blei mit 5,5 µg/g Kreatinin und 3,9 µg/l bestimmt. Die Differenz zwischen Gesamtbleigehalt und der Konzentration an anorganischem Blei ergibt, dass ca. 50 % als organisches Blei ausgeschieden wurden (Vural und Duydu 1995).

In einer weiteren Studie wurden im Urin Gesamt-Blei (PbT-U), anorganisches Blei (PbI-U) und Aminolävulinsäure (ALA) untersucht. Betrachtet wurden 49 Beschäftigte einer Raffinerie, 50 Mechaniker und 35 Kontrollpersonen in Ankara (Türkei). Die Kontrollpersonen wurden nach dem Zufallsprinzip ausgewählt und lebten und arbeiteten im zentralen Teil der Stadt. Aus Tabelle 3 kann man ersehen, dass auch bei den vermutlich nur gegen Bleitetraalkyle exponierten Beschäftigten der Raffinerie unter Berücksichtigung der Kontrollen dieses zu 50 % in anorganisches Blei metabolisiert und mit dem Urin ausgeschieden wurde. Bei den Raffinerie-Beschäftigten und den Mechanikern wurde eine statistisch signifikant erhöhte ALA-Ausscheidung im Urin gemessen, was auf eine übermäßige ALA-Produktion durch die Blei-bedingte Hemmung der ALA-Dehydratase zurückzuführen ist (Duydu und Vural 1998).

**Tab. 3:** Blei im Urin von beruflich Exponierten und Kontrollpersonen in der Türkei (Duydu und Vural 1998)

**Parameter**	**Raffinerie-Beschäftigte** **(n = 49)**	**Motor-Mechaniker** **(n = 50)**	**Kontrollen** **(n = 35)**
Mittleres Alter (Jahre)	34	28	23
PbT-U (µg Pb/l)	105,52 ± 8,54	89,15 ± 6,95	51,58 ± 2,40
PbI-U (µg Pb/l)	72,33 ± 4,42	62,90 ± 3,75	46,66 ± 1,64
PbT-U (µg Pb/g Kreatinin)	91,12 ± 5,49	67,25 ± 6,05	50,52 ± 2,93
PbI-U (µg Pb/g Kreatinin)	65,82 ± 3,68	48,43 ± 4,14	45,94 ± 2,39
Kreatinin (µg/l)	1,11 ± 0,07	1,63 ± 0,11	1,14 ± 0,05

PbT-U: Gesamtblei im Urin; PbI-U: anorganisches Blei im Urin

**Berechnung der Aufnahme und Ausscheidung aus der Studie von Zhang et al. (**1994**)**: Benzindepot-Beschäftigte nehmen bei einer Expositionskonzentration von 40,9 µg Pb/m^3^, einem Atemvolumen während einer achtstündigen Arbeitszeit von 10 m^3^ und einer kalkulierten inhalativen Retention von 30 % 120 µg Bleitetraethyl (als Blei berechnet) auf. Die Ausscheidung an Gesamtblei im Urin betrug 58 µg/l, davon abgezogen der Wert der Kontrollen von 16,9 µg/l und multipliziert mit 2, da von zwei Litern Gesamturin ausgegangen wird, berechnet sich eine Ausscheidung von ca. 80 µg Blei im Urin. Das entspricht ca. 67 % der retinierten Menge, 33 % werden mit den Faeces ausgeschieden oder verbleiben im Körper. Wie man der Tabelle 2 entnehmen kann, würde sich der prozentuale Anteil des nicht im Urin ausgeschiedenen Bleis bei höheren Expositionskonzentrationen erhöhen. 

Das Verhältnis Diethylblei zu anorganischem Blei im Urin lässt sich ebenfalls aus der Tabelle 2 ablesen. Bei einer Expositionskonzentration von 40,9 µg/m^3^ beträgt die Gesamtblei-Ausscheidung 58 µg/l, nach Abzug des Kontrollwerts (16,9 µg/l) und des Wertes für Diethylblei (18,8 µg/l), werden ca. 20 µg anorganisches Blei/l ausgeschieden. Damit sind bei dieser Expositionskonzentration ca. 50 % der Gesamtblei-Ausscheidung im Urin Blei-Ionen. 

Ein ca. 50%iger Anteil an Blei-Ionen an der Gesamtblei-Ausscheidung im Urin wird von den Daten der chronisch gegen Bleitetraalkyle belasteten Tankstellen-Beschäftigten in der Publikation von Vural und Duydu (1995) bestätigt.

#### 3.1.2 Tier

Eine einstündige Exposition an fünf aufeinanderfolgenden Tagen gegen 1100 mg Bleitetraethyl/m^3^ (700 mg Pb/m^3^) führte bei Charles-River-Ratten (n = 4) zu folgenden Bleigehalten (pro 100 g Trockengewicht): Knochen 0,44 mg, Gehirn 1,0 mg, Fett 0,62 mg, Niere 7,4 mg, Leber 8,6 mg, Lunge 5,14 mg und Milz 13,0 mg. Die exponierten Tiere verendeten drei Tage nach der letzten Exposition. Eine Exposition gegen 900 mg Bleitetramethyl/m^3^ (700 mg Pb/m^3^) führte bei Ratten (n = 4) zu folgenden Bleigehalten (pro 100 g Trockengewicht): Knochen 0,73 mg, Gehirn 1,72 mg, Fett 0,16 mg, Niere 5,45 mg, Leber 4,0 mg, Lunge 3,93 mg und Milz 5,5 mg (k. w. A.; Haskell Laboratories 1992 b).

Nach Verbrennung von Benzin mit einem Gehalt von 0,65 g ^210^Pb-Bleitetraethyl/l (als Blei berechnet) oder 0,5 g/l wurde das Auspuff-Aerosol zur Exposition von Ratten verwendet (Boudene et al. 1977; Morgan und Holmes 1978). Da es sich bei dem Aerosol zu einem hohen Anteil um anorganisches Blei handelt, das durch die Verbrennung des Bleitetraethyls entstanden ist, werden diese Studien nicht zur Bewertung der Toxikokinetik herangezogen.

Je 20 junge männliche SD-Ratten (entwöhnt und zehn Tage akklimatisiert, vermutlich 31. Postnataltag, Gewicht 87 g) erhielten 13 Wochen lang an fünf Tagen pro Woche per Schlundsonde 0; 0,05; 0,1; 0,2; 0,5 oder 1,0 mg Triethylbleiacetat/kg KG und Tag oder 200 mg **Bleiacetat**/kg KG und Tag. Von je fünf Ratten pro Gruppe wurde der Bleigehalt im Gehirn ermittelt. Hier waren die Gehalte an Pb^2+^ nach Gabe von 0,05 mg Triethylbleiacetat/kg KG oder 200 mg **Bleiacetat**/kg KG ähnlich hoch. Gesamtblei-Messungen im Gehirngewebe am 91. Tag zeigten einen Gehalt von 0,18 µg/g bei den Kontrolltieren, von 1,22 µg/g bei den Tieren der niedrigsten Dosisgruppe und von 8,48 µg/g bei den Tieren der höchsten Dosisgruppe der organischen Bleiverbindung. Trotz der hohen Dosis von 200 mg **Bleiacetat**/kg KG reicherte sich im Gehirn nur 0,95 µg Pb^2+^/g an (siehe Abschnitt 5.2.2; IARC 2006; Yagminas et al. 1992). Nach oraler Gabe von Bleitetraethyl verbleibt mehr Blei im Gehirn als nach oraler Aufnahme anorganischer Bleiverbindungen.

Eine tägliche sechsmonatige Bleitetraethyl-Schlundsondengabe von 6 µg/kg KG (bezogen auf Blei) an je zwei männliche und weibliche Rhesus-Affen (wild gefangen) führte zu einer geringen Erhöhung der Bleikonzentration im Blut. Vom Kontrollwert vor der Exposition von 26 µg/l Blut stieg die Bleikonzentration im Blut in den ersten vier Wochen kaum an, nach 36–57 Tagen auf 62,6 µg/l und betrug im Mittel nach 71–176 Tagen 43,9 µg/l (Heywood et al. 1979). Auffällig ist, dass die Bleikonzentrationen in Urin und Faeces nach der Bleitetraalkyl-Gabe in den ersten vier Wochen im Vergleich zu den Werten vor der Applikation sanken und im Blut nur eine sehr geringe Zunahme an Blei beobachtet wurde. Die Hauptmenge wurde bei den männlichen Tieren in die Knochen eingelagert.

Mit dem Trinkwasser, das Bleitetraethyl in Konzentrationen von 0; 0,5; 1,0; 2,0 oder 10 mg/l (als Blei berechnet, mit Tween 80 als Emulgator) enthielt, erhielten je zehn männliche Swiss-Cross-Mäuse drei Wochen lang eine tägliche Bleitetraethyl-Dosis von ca. 0; 0,1; 0,2; 0,4 oder 1,0 mg/kg KG (bezogen auf Blei). In der Niere und der Leber wurden ab 0,1 mg/kg KG mit 6,6 bzw. 5,3 µg/g Feuchtgewicht statistisch signifikant erhöhte Bleigehalte beobachtet (Blakley et al. 1980).

Je fünf männliche Sprague-Dawley-Ratten erhielten 13 Wochen lang an fünf Tagen pro Woche per Schlundsonde 0; 0,2 oder 2 mg Bleitetraethyl/kg KG und Tag (in Wasser gelöst mit 1 % Emulphor) oder nur in Wasser gelöst 0; 0,2; 1,0 oder 2 mg Triethylbleiacetat/kg KG und Tag oder 200 mg **Bleiacetat**/kg KG und Tag. Die höchsten Konzentrationen der organischen Bleiverbindungen fanden sich in der Leber, gefolgt von Niere und Blut. Das Blei nach Bleiacetat-Gabe reicherte sich hauptsächlich in den Nieren an. In weiteren Organen und Knochen wurde der Bleigehalt nicht untersucht. Triethylblei war der Hauptmetabolit der organischen Bleiverbindungen (siehe Abschnitt 5.2.2; Franklin et al. 1987).

Nach oraler Applikation von organischen Bleiverbindungen werden bezogen auf Gesamtblei ca. 2/3 mit den Faeces und 1/3 mit dem Urin ausgeschieden (k. w. A.; BUA 1994).

Eine siebentägige intravenöse Gabe von 6 mg Bleitetraethyl/kg KG an je zwei weibliche und männliche Rhesusaffen (wild gefangen) führte zu erhöhten Bleigehalten in den folgenden Organen (Nassgewicht): Gehirn 7,2 µg/g, Leber 48,0 µg/g, Niere 50,0 µg/g und Knochen 15,6 µg/g. Alle Gehalte waren nach einer 28-tägigen Nachbeobachtungsphase deutlich gesunken, außer im Knochen (17,0 µg/g). Nach intravenöser Gabe von 6 mg Bleitetramethyl/kg KG lagen die Bleigehalte in den Organen deutlich niedriger: Gehirn 2,0 µg/g, Leber 24,5 µg/g, Niere 10,3 µg/g und Knochen 1,3 µg/g. Auch hier wurde mit 3,4 µg/g eine Zunahme an Blei im Knochen nach 28 Tagen Nachbeobachtung gemessen (Heywood et al. 1978).

Nach intravenöser Injektion von 31 µmol Bleitetraethyl/kg KG (10 mg/kg KG) in Ethanol wurden die Triethylblei-Konzentrationen in verschiedenen Geweben von Ratten bestimmt. Nach drei Tagen waren die Triethylblei-Konzentrationen in Leber, Blut und Niere am höchsten und sanken in den folgenden Tagen wieder. Im Gehirn wurde die höchste Triethylblei-Konzentration am 8. Tag gemessen. Nach Injektion von 20 mg Bleitetraethyl/kg KG wurden 24 Stunden später im Blut 0,086 µmol Triethylblei/g (ca. 25 µg/g) und 0,174 µmol Gesamtblei/g (ca. 36,0 µg/g), in der Leber 0,065 µmol Triethylblei/g (ca. 19 µg/g) und 0,147 µmol Gesamtblei/g (ca. 30,5 µg/g) sowie im Gehirn 0,045 µmol Triethylblei/g (ca. 13,2 µg/g) gemessen. Bei einer intravenösen Injektion von 30,4 µmol Triethylbleichlorid/kg KG (ca. 10 mg/kg KG) war Triethylblei eine Woche stabil (Bolanowska 1968).

Nach intravenöser Injektion von 12 mg Bleitetraethyl/kg KG an drei Kaninchen waren von der 24 Stunden später im Urin bestimmten Bleimenge 69 % Diethylblei, 27 % anorganisches Blei und 4 % Triethylblei. Dieses Verhältnis wurde auch in den folgenden sieben Tagen beobachtet. In den Faeces wurde fast ausschließlich anorganisches Blei gefunden (85–95 %). In Leber, Niere und Blut lagen 84, 68 bzw. 59 % der jeweiligen Blei-Gesamtmenge als Triethylblei vor. Mit den Faeces wurde deutlich mehr Blei ausgeschieden als im Urin (Arai et al. 1981). Diese Publikation liegt in japanischer Sprache mit englischen Abbildungen, Tabellen und Abstract vor.

Nach einer einmaligen intravenösen Injektion von 12 mg Bleitetraethyl/kg KG an zwei Kaninchen fanden sich 24 Stunden später 8 % der applizierten Bleimenge in der Gallenflüssigkeit fistulierter Tiere. Es lag fast vollständig (97 %) als Diethylblei vor. Im Blinddarm-Faeces nicht fistulierter Tiere wurden 12 % des injizierten Bleis 24 Stunden nach der Gabe gemessen. Hier kam fast ausschließlich anorganisches Blei vor (Arai et al. 1983). Diese Publikation liegt in japanischer Sprache mit englischen Abbildungen, Tabellen und Abstract vor.

Nach einmaliger intravenöser Injektion von 9,9 mg Bleitetramethyl/kg KG (7,7 mg Pb/kg KG) in die Ohrvene von drei Kaninchen bestand die im Urin innerhalb von sieben Tagen ausgeschiedene Bleimenge zu 73 % aus Dimethylblei, 19 % aus Trimethylblei, 6 % aus Pb^2+^ und 2 % aus Bleitetramethyl. Bei drei Kaninchen waren die entsprechenden Werte nach einmaliger Injektion von 39,7 mg Bleitetramethyl/kg KG (30,8 mg Blei/kg KG) 67 % Dimethylblei, 14 % Trimethylblei, 17 % Pb^2+^ und 1 % Bleitetramethyl. In den Faeces fand sich ausschließlich anorganisches Blei. Von der gesamten injizierten Bleimenge wurden in den sieben Tagen 1–3 % mit dem Urin und 7–19 % mit den Faeces ausgeschieden. Nach sieben Tagen wurden die höchsten Bleigehalte in der Leber gemessen, gefolgt von Niere > Muskel > Gehirn > Herz > Rückenmark > Lunge, Pankreas, Milz > Knochenmark. Der Bleigehalt in der Leber setzte sich aus etwa 89 % Trimethylblei, etwa 4 % Dimethylblei und etwa 7 % Pb^2+^ sowie in den Nieren aus etwa 85 % Trimethylblei und etwa 15 % Pb^2+^ zusammen. Die Bleimenge im Gehirn bestand aus etwa 87 % Trimethylblei und 13 % Pb^2+^. In keinem Organ wurde Bleitetramethyl nachgewiesen (Arai und Yamamura 1990). Die Gesamtbleiausscheidung von 1–3 % im Urin nach sieben Tagen erscheint sehr niedrig.

Triethylneopentoxyblei (C_2_H_5_)_3_Pb[OCH_2_C(CH_3_)_3_] wurde nach Injektion von 10 mg/kg KG (5,4 mg Pb/kg KG) an Kaninchen nur zu 4 % im Urin und zu 68 % in den Faeces nach sieben Tagen ausgeschieden. Wie auch bei Tetra- und Triethylblei waren die Hauptmetaboliten im Urin Diethylblei und in den Faeces anorganisches Blei (Arai et al. 1998).

Ratten und Mäuse erhielten intraperitoneal eine unbekannte Menge Bleitetraethyl. Die maximale Konzentration in Blut und Leber war nach ein bis drei Stunden erreicht. In der Niere fanden sich 11 % der verabreichten Menge nach 20 Stunden. Die Halbwertszeit im Gesamtkörper betrug bei Mäusen drei Tage und bei Ratten weniger als einen Tag. Nach intraperitonealer Triethylblei-Verabreichung konnten folgende Halbwertszeiten bestimmt werden: zwei Tage im Gesamtkörper und drei bis fünf Tage im Blut der Maus, zehn Tage im Blut, 15 Tage in Leber und Niere und sieben bis acht Tage im Gehirn der Ratte (k. w. A.; ECHA 2022 b).

Bei neonatalen Fischer-344-Ratten, denen am 5. Postnataltag Triethylbleichlorid subkutan in Dosierungen von 0; 4,5 oder 9 mg/kg KG (ca. 0; 2,8; 5,6 mg Pb/kg KG) injiziert wurde, betrugen am 22. Postnataltag die Konzentrationen im Blut 70, 150 bzw. 350 µg/l und im Gehirn 0,2; 1,5 bzw. 9,0 µg/g Trockengewicht. Nach 40 Tagen lag die Konzentration im Gehirn unter der Nachweisgrenze von 2 ng/ml (Booze und Mactutus 1990).

### 3.2 Metabolismus

Bleitetraethyl wird in der Leber durch Cytochrom-P450 oxidativ dealkyliert. Der Metabolit Trialkylblei verteilt sich im ganzen Körper und ist für die neurotoxische Wirkung verantwortlich (Bolt 1996; Henschler 1972 a; Tenenbein 1997).

Nach intravenöser Gabe von Bleitetraethyl an Kaninchen fand sich fast ausschließlich Diethylblei in der Gallenflüssigkeit, das später als anorganisches Blei mit den Faeces ausgeschieden wurde (Arai et al. 1983).

Alkoholkonsum beeinflusste die Aufnahme von organischen Bleiverbindungen, da die Blutbleikonzentrationen weniger stark anstiegen im Vergleich mit Exponierten ohne Alkoholkonsum. Das gibt einen Hinweis darauf, dass die Enzym-vermittelte Metabolisierung des Bleitetraethyls in der Leber durch Alkohol verändert wurde. Alkohol beeinflusste nicht die Aufnahme von anorganischen Bleiverbindungen (McGrail et al. 1995).

Bleitetraethyl kann letztendlich zu anorganischem Blei oxidiert werden und wird dann in den Knochen gespeichert oder ausgeschieden (IARC 2006).

### 3.3 Fazit

Bleitetraalkyle sind aufgrund ihrer lipophilen Eigenschaften gut durch die Haut resorbierbar (Aufnahme ca. 6 %) und werden inhalativ gut resorbiert und zu ca. 30 % retiniert. Bei oraler Aufnahme werden Bleialkyle beim Tier über den Magen-Darm-Trakt resorbiert. Bleitetraalkyle werden zum Teil unverändert abgeatmet. Bleitetraalkyle reichern sich deutlich stärker im Gehirn an als anorganisches Blei. Nach erfolgter Dealkylierung im Körper ist davon auszugehen, dass die entstandenen anorganischen Blei-Ionen in gleicher Weise wie direkt verabreichtes anorganisches Blei reagieren. Es kann damit zu einer Anreicherung im Knochen kommen. Die Ausscheidung erfolgt als Dialkylblei und als anorganisches Blei mit dem Urin und fast ausschließlich als anorganisches Blei mit den Faeces.

## 4 Erfahrungen beim Menschen

Im Unterschied zu anorganischen Bleiverbindungen traten nach akuten Bleitetraalkyl-Vergiftungen Psychosen wie Manie und Verwirrung sowie Schizophrenie und Delirium am Arbeitsplatz auf. Es kam zu Auswirkungen auf die vitalen Funktionen wie zu niedriger Blutdruck und Körpertemperaturabsenkung. Des Weiteren wurde häufig Schlaflosigkeit und Anorexie beschrieben (Tenenbein 1997).

### 4.1 Einmalige Exposition

Es sind zahlreiche Fälle akuter Vergiftung mit Bleitetraethyl beschrieben. Symptome einer schweren akuten Vergiftung sind akustische und optische Halluzinationen, Krampfanfälle, motorische Störungen und Sprachstörungen sowie Delirium und Koma (Henschler 1972 a).

Ein 13-jähriger Junge, der versehentlich einen „Mundvoll“ 80–90%iges Bleitetraethyl zu sich nahm, bekam innerhalb von Minuten diffuse Bauchschmerzen und erbrach sich. Im Verlauf der nächsten 48 Stunden entwickelte er progressiven Tremor, Halluzinationen und allgemeine Schwäche. Er wurde medikamentös behandelt und erhielt u. a. am 3. Tag nach der Vergiftung für fünf Tage EDTA. Die Behandlung war nach 91 Tagen erfolgreich und er zeigte keine Symptome mehr. Die Blutbleikonzentrationen betrugen am 3. Tag und am 32. Tag 620 µg/l und am 62. Tag 510 µg/l. Die Urinwerte lagen am 3. Tag bei 2700 µg/l, am 10. Tag bei 1600 µg/l und am 51. Tag bei 51 µg/l (Wills et al. 2010).

Nach versehentlicher inhalativer und dermaler Aufnahme einer unbekannten Menge von Bleitetramethyl wurden im Blut einer Patientin 1370 µg Blei/l und fünf Tage später 1500 µg/l gemessen. Die Serumwerte betrugen bei der Patientin 703 µg/l und fielen auf 300 µg/l nach zwei Monaten ab. Im Urin stieg die Bleikonzentration von 12 µg/l nach zwei Monaten auf 1100 µg/l nach drei Monaten an. Die Patientin war mehrwöchig bewusstlos. Es blieben massive Funktionseinschränkungen des zentralen und peripheren Nervensystems auch vier Jahre nach der Vergiftung bestehen. Der ebenfalls exponierte Ehemann zeigte weniger ausgeprägte Symptome, was durch die niedrigere Bleiaufnahme begründet ist, so betrug die Bleikonzentration im Serum des Ehemannes 104 µg/l (Schiele et al. 1995).

Nach akuter Vergiftung mit Bleitetramethyl (k. w. A.) wurden keine neurologischen Symptome beobachtet, obwohl eine Blutbleikonzentration von 810 µg/l vorlag. Die Autoren schließen daraus, dass Bleitetramethyl im Vergleich mit Bleitetraethyl weniger neurotoxisch ist (Gething 1975; Greim 1995).

### 4.2 Wiederholte Exposition

Nach chronischer Exposition („Schnüffler“) wurden Erregbarkeit, Depression, Kopfschmerz, niedriger Blutdruck, Neurasthenie, Tremor, Ataxie, Chorea und Halluzinationen beobachtet. Patienten, die bereits seit der Jugend über viele Jahre Benzin schnüffelten, waren oft geistig stark zurückgeblieben. Die Autopsie zeigte Gehirnödeme, Nekrosen von Nervenzellen im Hippocampus, Verlust von Purkinjezellen im Cerebellum und Demyelinisierung des Pons sowie Kleinhirnatrophie und ein insgesamt reduziertes Gehirngewicht (Bolt 1996).

In einem Betrieb der Bleialkyl-Herstellung in Deutschland mit einer mittleren Expositionskonzentration an Bleitetraethyl von 0,6–43,1 µg/m^3^, berechnet als Blei, und einer Expositionszeit der Beschäftigten von 14,6 ± 6,2 Jahren wurden neuropsychologische Tests bei 38 Beschäftigten durchgeführt. Eine Korrelation zwischen erhöhten Trimethylblei-Werten im Urin (Mittelwert 2,43 ± 2,37 ng/mg Kreatinin) und eingeschränkten Fähigkeiten im Zahlen-Symbol-Test wurde beobachtet (partielle Korrelation). Es wurden weiter keine Effekte festgestellt (Bolt 1996; Greim 1994; Seeber et al. 1990). In einer Studie zur Messmethodik ergaben Messungen an diesen Arbeitsplätzen Expositionskonzentrationen von Bleitetraethyl bis zu 46 µg/m^3^ und von Bleitetramethyl bis zu 7,5 µg/m^3^, jeweils als Blei berechnet (Backes et al. 1989). Da in diesem Betrieb auch Blei geschmolzen wurde, ist von einer Mischexposition mit anorganischem Blei auszugehen.

Bei 227 Beschäftigten, die in einem Benzindepot gegen eine mittlere Bleitetraethyl-Konzentration von 84,8 ± 34,3 µg/m^3^ bezogen auf Blei exponiert waren, wurden klinische Parameter untersucht und verglichen mit 36 geringer belasteten Verkehrspolizisten sowie 342 unbelasteten Büroangestellten. Bei den Benzindepot-Beschäftigten trat bei Expositionen gegen 50–69 µg/m^3^ (als Blei berechnet, n = 168) bei 28 Personen (16,67 %) und damit mit statistisch signifikant erhöhter Häufigkeit Tremor (Hände, Zunge, Augenlider) auf. In der Gruppe (n = 27) mit einer Bleitetraethyl-Exposition gegen 30–49 µg/m^3^ als Blei berechnet, lag die Häufigkeit des Auftretens von Tremor mit zwei Betroffenen (7,41 %) im Bereich der Kontrollgruppe (21 Personen, 6,14 %) (Greim 1994; Zhang et al. 1994). Das Auftreten von Tremor wird als sehr schwerwiegender Effekt betrachtet, da diesem Effekt bereits neurotoxische Störungen vorausgehen, vor denen ebenfalls geschützt werden muss.

#### 4.2.1 Studien seit dem letzten Nachtrag zu Bleitetraethyl aus dem Jahr 1994

Die Untersuchung der Blutbleikonzentration bei 42 Tankstellen-Beschäftigten, 47 Busfahrern und 47 Taxifahrern, jeweils aus dem Zentrum von Athen, ergab 56,4 µg/l; 58,8 µg/l bzw. 59,6 µg/l. Die Kontrollen (Krankenhaus-Mitarbeiter, n = 33) hatten einen Blei-Gehalt von 57,6 µg/l Blut. Das Elektroenzephalogramm ergab keine signifikanten Unterschiede zwischen den Kontrollpersonen und den Beschäftigten, sowie den Fahrern. Weitere Endpunkte wurden nicht untersucht (Kapaki et al. 1998).

Eine weitere Studie mit dem bei Kapaki et al. (1998) beschriebenen Kollektiv ergab bei den Tankstellen-Beschäftigten für geringe bis mäßige kortikale Atrophie ein statistisch signifikant erhöhtes Risiko mit einem Odds Ratio (OR) von 6,43 (95-%-KI: 1,46–28,27) und für das gesamte Kollektiv einen leichten Anstieg an kortikaler Atrophie in Bezug auf die Blutbleikonzentration mit einem OR von 1,4 (95-%-KI: 1,01–2,05). Rauchen, Alter und die Dauer der Exposition zeigten keinen Einfluss auf das Auftreten von Atrophie (Varelas et al. 1999). Es ist davon auszugehen, dass Tankstellen-Beschäftigte einer höheren Belastung durch organische Bleiverbindungen ausgesetzt sind als Busfahrer und Taxifahrer.

In einer Firma in China, die gewebte Kunststoffbeutel herstellte, zeigten sich bei einer Reihe von Mitarbeitern auffällige psychische Symptome. Dabei handelte es sich um Sprachstörungen, Aggressionen sowie akustische und visuelle Halluzinationen. Zur Behandlung ins Krankenhaus wurden 16 Beschäftigte aufgenommen. Schwere Fälle („acute and heavy patients“) wurden sogar sediert. Sie bekamen je nach Ausprägung der Neurose Aloperidin oder Midazolam. Die Patienten litten unter Übelkeit, Kopfschmerzen, Hyperspasmie und Schwäche der Gliedmaßen, Sprachstörungen, nonverbalen akustischen Halluzinationen („acouasm“), Psychose, Ängsten und Delirium. Die Patienten hatten Blutbleikonzentrationen von 417–772 µg/l und Urinbleikonzentrationen von 168–2020 µg/l. Die Bleikonzentrationen in Blut und Urin korrelierten nicht mit der Schwere der psychischen Symptome der Patienten. Die Aktivitäten der Enzyme Hydroxybutyratdehydrogenase, Laktatdehydrogenase, Kreatinkinase und Kreatinkinase-MB (Kreatininkinase der Herzmuskelzellen) waren bei allen Patienten statistisch signifikant erhöht. Im Blut wurde etwa bei der Hälfte der Patienten ein niedriger Kaliumwert beobachtet. Eine bakterielle Infektion konnte ausgeschlossen werden. Auf der Suche nach dem Verursacher der medizinisch-psychiatrischen Symptome wurde das Material des Produktionsprozesses untersucht. „Weißöl“, das als Schmiermittel beim Weben mit der Hand verwendet wurde, enthielt 3,1–12,3 mg Bleitetraethyl/kg. Die Beschäftigten trugen keine Handschuhe, so dass eine Aufnahme durch die Haut sowie Hand-Mund-Kontakt anzunehmen ist. In der Luft konnten weder Blei, Zinn noch flüchtige organische Substanzen nachgewiesen werden (Zhang et al. 2016).

In einer Studie in Nigeria war bei 35 Bleitetraethyl-Anwendern („lead handlers“) und 40 Tankwarten („fillers“) im Vergleich mit 36 unbelasteten Grundschullehrern die FEF_25–75_ (mittlere Atemstromstärke nach Ausatmung von 25–75 % der forcierten Vitalkapazität) statistisch signifikant verringert. Zudem waren die Exponierten häufiger von produktivem Husten, Kopfschmerzen und Schlafstörungen betroffen. Die Autoren interpretieren die Daten dahingehend, dass die Resorption von organischen Bleiverbindungen die Lunge schädigt, was zu einer verstärkten Aufnahme über die Lunge führen kann (Afolabi et al. 1999). Die Studie liegt nur als Abstract vor. Expositionskonzentrationen sind nicht angegeben.

#### 4.2.2 Studien mit Mischexposition gegen organische und anorganische Bleiverbindungen

Den nachfolgend beschriebenen Studien liegen Messungen in einem Bleitetraethyl-produzierenden Betrieb in New Jersey, USA, zugrunde. Der Herstellungsprozess des Bleitetraethyls beinhaltete das Schmelzen von Bleibarren, und bei den meisten Beschäftigten lag eine Mischexposition gegen anorganisches und organisches Blei vor (Schwartz et al. 1993).

In einer Studie, die 222 mit Bleitetraethyl, Bleitetramethyl und **anorganischem Blei** belastete männliche Beschäftigte mit einer mittleren Expositionszeit von 13,3 ± 9,5 Jahren aus einem Bleitetraethyl- und Bleitetramethyl-Produktionsbetrieb in den USA umfasste, beobachteten Balbus et al. (1997) eine mittlere Blutbleikonzentration von 205 ± 100 µg/l (Bereich 10–510 µg/l), eine mittlere Spitzen-Blutbleikonzentration von 317 µg/l (Bereich 0–730 µg/l) und eine mittlere Urinbleikonzentration von 143 ± 130 µg/l (Bereich 0–1035 µg/l) der exponierten Beschäftigten. Der Betrieb wurde nach Arbeitsbereichen in 29 Zonen unterteilt und dort die Expositionen ermittelt (Schwartz et al. 1993). Es wurden Mittelwerte von organischen Bleiverbindungen in einem Bereich von 4–119 µg Blei/m^3^ und anorganischen Bleiverbindungen in einem Bereich von 1–56 µg Blei/m^3^ gemessen. Die individuellen kumulativen Bleiexpositionen wurden berechnet und die Beschäftigten danach in vier Gruppen eingeteilt: ≤ 100, 101–530, 531–1330 und > 1330 µg/m^3^ × Jahre sowie nach Expositionsdauer: ≤ 2,1; 2,2–9,5; 9,6–21,45 und > 21,45 Jahre. Die niedrig exponierte Gruppe diente in den folgenden Auswertungen als Vergleichsgruppe. Als Confounder wurden Alter, ethnische Zugehörigkeit, Alkoholkonsum und intellektuelle Fähigkeiten, ermittelt mit dem Wortschatz-Test (“vocabulary score“), berücksichtigt. Da das Alter einen großen Einfluss zeigte, wurden Personen < 35 Jahre ausgeschlossen. Als weitere Confounder waren Expositionen gegen Ethylendichlorid, Ethylendibromid, Ethylchlorid und kommerzielle Lösungsmittel bestimmt worden. Diese Substanzen gelten nach Meinung der Autoren als weniger neurotoxisch im Vergleich zu Blei und wurden daher, ebenso wie das Bildungsniveau und der kumulative Zigarettenkonsum, die sich nicht als Störvariablen der Zusammenhänge zwischen Bleiexposition und Testleistung herausstellten, letztlich in den Modellen nicht berücksichtigt. Nach den folgenden Verfahren wurden die Beschäftigten untersucht: vom Prüfer durchgeführte neurologische Verhaltenstests, computergestützte neurologische Verhaltenstests und neuropsychiatrische Fragebögen, einschließlich des Minnesota Multiphasic Personality Inventory, Geruchsidentifikationstests und Messung der peripheren Vibrationsschwelle im Großzeh. Nach Berechnung mit dem Student-t-Test konnten Unterschiede zu den Kontrollen bei Studienteilnehmern festgestellt werden, die 2,2–9,5 Jahre beschäftigt waren oder einer Bleiexposition von 101–530 µg/m^3^ × Jahre ausgesetzt waren. Eine statistisch signifikante Veränderung (p < 0,045) zeigte diese moderat belasteten Gruppen nur im Digit Symbol-Test. Ab einer Beschäftigungsdauer von 9,6 Jahren traten statistisch signifikante Unterschiede in der verbalen Gedächtnisleistung und in neuropsychiatrischen Tests auf. Manuelle Geschicklichkeit war statistisch signifikant eingeschränkt ab einer Beschäftigungsdauer von 21,45 Jahren. Eine kumulative Bleibelastung führte bereits ab 531 µg/m^3^ × Jahre zu statistisch signifikanten Einschränkungen bei manueller Geschicklichkeit und verbaler Gedächtnisleistung. Bei Betrachtung aller 192 adjustierten Unterschiede der jeweiligen Gruppenmittelwerte aus 32 Tests zeigten 51 (27 %) schlechtere neurologische Fähigkeiten der Exponierten im Vergleich mit den Kontrollen. Anhand von Trendtests wurden bei 15 (23 %) der neuropsychologischen Tests geringere Leistungen mit zunehmender Exposition festgestellt. Dabei waren einzelne Testleistungen in der Gruppe der am höchsten exponierten Beschäftigten um 5–22 % niedriger als bei der Vergleichsgruppe. Der Versuch, die Effekte durch organische und anorganische Bleiverbindungen getrennt zu modellieren, war nicht möglich, da unzureichende Stichprobendaten und eine zu geringe Anzahl von Studienteilnehmern für eine Korrelation zwischen der Exposition gegen organische und anorganische Bleiverbindungen vorlagen (Schwartz et al. 1993). Da in dieser Studie Mischexpositionen gegen organische und anorganische Bleiverbindungen vorhanden waren und keine Zuordnung der Effekte zu entsprechenden Bleibelastungen/-Expositionen möglich war, können die Ergebnisse nicht zur Ableitung eines Grenzwertes herangezogen werden.

Die weitere Auswertung der Daten des bei Schwartz et al. (1993) beschriebenen Kollektivs mit Expositionen gegen organische und anorganische Bleiverbindungen zeigte eine ansteigende Urinbleikonzentration von 39 µg/l im Jahr 1965 auf 61 µg/l im Jahr 1985 und eine Abnahme auf 52 µg/l im Jahr 1990. Alkoholkonsum beeinflusste die Aufnahme von organischem Blei, da die Blutbleikonzentrationen bei Exponierten mit Alkoholkonsum weniger stark anstiegen im Vergleich mit Exponierten ohne Alkoholkonsum (McGrail et al. 1995).

Die Auswertung der Untersuchungsergebnisse einer Fallserie von Patienten eines Krankenhauses umfasste 57 männliche und eine weibliche Beschäftigte des bei Schwartz et al. (1993) beschriebenen Bleitetraethyl- und Bleitetramethyl-Produktionsbetriebes, die gegen organische und anorganische Bleiverbindungen exponiert waren. Die mittlere Blutbleikonzentration, auf Lebenszeit berechnet, betrug 261 ± 91 µg/l (n = 48), die mittlere Urinbleikonzentration 51,2 ± 18,8 µg/l (n = 48) und die mittlere aktuelle Blutbleikonzentration 194 ± 65 µg/l (n = 39). Das mittlere Alter betrug 44,5 ± 7,1 Jahre und die mittlere Beschäftigungszeit 14,4 ± 7,4 Jahre. Nach den folgenden Verfahren wurden die Patienten untersucht: vom Prüfer durchgeführte neurologische Verhaltenstests, computergestützte neurologische Verhaltenstests und neuropsychiatrische Fragebögen. Die häufigsten Symptome waren Gedächtnisverlust (74 %), Gelenkschmerzen (56 %), Schlafstörungen (54 %), Reizbarkeit (51 %), Parästhesien und Müdigkeit (jeweils 49 %), Alpträume (35 %), Unausgeglichenheit (28 %), Kopfschmerzen und Depressionen (je 21 %), Muskelschmerzen (12 %) sowie Schwäche (11 %). Nur zwei der Patienten waren zum Zeitpunkt der Untersuchung beschwerdefrei. Bei den Beschäftigten mit PNS-Symptomen (n = 31) wurde zusätzlich die Nervenleitfähigkeit untersucht. Bei elf von ihnen trat Polyneuropathie auf. Zumindest eine Auffälligkeit wurde bei 22 der 31 untersuchten Patienten gefunden. Keine Auffälligkeiten zeigten neun Patienten (Mitchell et al. 1996). Es konnte nicht zwischen Exposition gegen organische und anorganische Bleiverbindungen unterschieden werden.

Eine weitere Studie befasst sich mit dem bei Schwartz et al. (1993) beschriebenen Kollektiv. Ausgewertet wurden hier die Ergebnisse der Methode „simple visual reaction time“ (SVRT). Bei diesem Test wird die Zunahme an Fehlern und die Reaktionszeit bestimmt. Bei Berücksichtigung der Standardabweichungen oder Varianz bei der Interpretation der Ergebnisse der SVRT-Methode, da sich die Neurotoxizität in einem Anstieg an Fehlern zeigen kann, fand sich ein statistisch signifikanter Effekt ab einer aktuellen Gesamt-Blutbleikonzentration von 300 µg/l. Eine Korrelation mit dem Alter der Exponierten zeigte hier ebenfalls statistische Signifikanz. Dieser Effekt korrelierte nicht mit den aktuellen Bleiexpositionskonzentrationen an organischen oder anorganischen Bleiverbindungen und den kalkulierten kumulativen Bleikonzentrationen in Blut oder Urin. Die Auswertung der Daten nach Exposition ausschließlich gegen organische Bleiverbindungen war nicht möglich (Balbus et al. 1997; Schwartz et al. 1993). 

Bei 535 Beschäftigten einer ehemals Bleitetraethyl-produzierenden Fabrik in New Jersey, USA, die seit einer mittleren Zeitspanne von 16 Jahren nicht mehr gegen organische und anorganische Bleiverbindungen exponiert waren, wurden über einen Zeitraum von vier Jahren neurologische Tests durchgeführt und mit dem Bleigehalt der Tibia korreliert. Bei ehemaligen Blei-Arbeitern lag der mittlere „Spitzen-Tibia-Bleiwert“, berechnet aus der aktuellen Höhe und der expositionsfreien Zeit, bei 22,6 ± 16,5 μg Pb/g Knochenmineral (Bereich –2,2–98,7 µg/g [sic]). Die mittleren Blutbleikonzentrationen betrugen 46 ± 26 µg/l (Bereich 10–200 µg/l). Verglichen mit einer Kontrollgruppe (n = 118) schnitten die bleibelasteten Beschäftigten in drei Tests (visuell-konstruktive Fähigkeit, verbales Gedächtnis und Lernen) über die Jahre schlechter ab. Eine hohe Tibia-Bleibelastung korrelierte mit einer Abnahme der kognitiven Fähigkeiten in sechs Tests (verbales Gedächtnis und Lernen, visuelles Gedächtnis, Ausführungsfähigkeit und manuelle Geschicklichkeit) (Schwartz et al. 2000).

Bei 543 Beschäftigten einer ehemals Bleitetraethyl-produzierenden Fabrik in New Jersey, USA, die seit einer mittleren Zeitspanne von 16 Jahren nicht mehr gegen organische und anorganische Bleiverbindungen exponiert waren, lag der „Spitzen-Tibia-Bleiwert“ bei –2,2–105,9 μg Pb/g Knochenmineral [sic]. Es wurden 20 neurologische Tests durchgeführt und mit dem „Spitzen-Tibia-Bleiwert“ korreliert. In einer früheren Untersuchung dieses Kollektivs mit aktueller Bleibelastung konnte ein klarer Zusammenhang zwischen hohen Tibia-Bleikonzentrationen und einem schlechteren Abschneiden bei 11 von 20 der neurologischen Tests gezeigt werden. Nach dieser langen Zeitspanne ohne Exposition gegen organische Bleiverbindungen fiel das Ergebnis nach einer Adjustierung (Alter, Bildungsstand, Depressions-Status und den Test durchführende Person) noch bei 7 von 20 der neurologischen Tests statistisch signifikant niedriger aus (visuelle Konstruktionsaufgaben, verbales Gedächtnis und Lernfähigkeit). Die Bestimmung des Genotyps des Apolipoproteins E bei 529 Beschäftigten ergab ε3ε3 (67,1 %), ε3ε4 (18,0 %), ε2ε3 (10,4 %), ε2ε4 (3,2 %), ε4ε4 (1,1 %) und ε2ε2 (0,2 %). Damit hatten 411 (77,7 %) kein ε4-Allel und 118 (22,3 %) mindestens ein ε4-Allel. Ein positiver Apolipoprotein E ε4-Status führte bei zwei neurologischen Tests mit steigenden Tibia-Bleikonzentrationen zu deutlich schlechteren Ergebnissen. Das heißt, dass sich die toxische Wirkung von Blei auf das ZNS verstärkt bei Personen, die ein ε4-Allel tragen (Stewart et al. 1999, 2002).

Die Untersuchung des Gehirns ließ bei dem bei Stewart et al. (1999, 2002) beschriebenen Kollektiv noch 16 Jahre nach Ende der beruflichen Exposition gegen organische Bleiverbindungen eine Korrelation zwischen hohen Tibia-Bleikonzentrationen und einer Zunahme von Läsionen in der weißen Substanz des Gehirns erkennen (Stewart et al. 2006).

Als Fazit der bei Stewart et al. (1999, 2002, 2006) und Schwartz et al. (2000) erhobenen Daten ziehen die Autoren, dass bei chronischer und kumulativer Bleibelastung durch organische oder anorganische Bleiverbindungen auch 16 Jahre nach der Exposition noch eine Einschränkung der kognitiven Fähigkeiten vorliegt. Die Autoren schließen daraus, dass die bisher ausschließlich dem Alterungsprozess zugeordneten Einschränkungen dieser Fähigkeiten durch die Bleibelastung in der Bevölkerung beschleunigt wurden (Stewart und Schwartz 2007).

**Fazit**: Die Blutbleikonzentrationen des hier betrachteten Kollektivs mit Mischexposition gegen anorganische und organische Bleiverbindungen liegen mit einer mittleren Konzentration von ca. 200 µg/l über dem BAT-Wert von 150 µg/l (Greiner et al. 2022). Da die Expositionsmittelwerte zeigen, dass die Belastung mit organischen Bleiverbindungen bei den meisten Beschäftigten höher war als gegen anorganisches Blei, können die beobachteten neurologischen Einschränkungen daher auf eine zumindest mit anorganischen Bleiverbindungen vergleichbare neurotoxische Wirkung der organischen Bleiverbindungen zurückgeführt werden.

### 4.3 Wirkung auf Haut und Schleimhäute

Reizeffekte an Haut- und Schleimhäuten sind nicht beschrieben (Greim 2001 a).

### 4.4 Allergene Wirkung

#### 4.4.1 Hautsensibilisierende Wirkung

Es liegen keine Daten vor.

Aus langjähriger beruflicher Erfahrung gibt es keine Hinweise auf signifikante sensibilisierende Wirkung des Bleitetramethyls und Bleitetraethyls (BUA 1994).

#### 4.4.2 Atemwegssensibilisierende Wirkung

Hierzu liegen keine Daten vor. 

### 4.5 Reproduktionstoxizität

Neue Daten liegen nicht vor.

#### 4.5.1 Fertilität 

Im Nachtrag aus dem Jahr 1994 wird über Störungen der Sexualfunktionen und der Spermatogenese bei Männern berichtet, die subakut bis chronisch sehr hohen, jedoch nicht genannten Bleitetraethyl-Konzentrationen ausgesetzt waren (Greim 1994).

#### 4.5.2 Entwicklungstoxizität 

Für Bleitetraethyl und Bleitetramethyl liegen zur Entwicklungstoxizität und Entwicklungsneurotoxizität keine belastbaren Daten beim Menschen vor. 

Im Addendum zur BAT-Begründung aus dem Jahr 2022 zu Blei und seinen anorganischen Verbindungen sind die Assoziationen zwischen entwicklungstoxischen Effekten (Geburtsparameter, anthropometrische Maße im Kindesalter) und der Bleikonzentration im pränatalen maternalen Blut oder Nabelschnurblut ausführlich dargestellt. Für die entwicklungsneurotoxische Wirkung von Blei gibt es ausreichend konsistente Evidenz aus prospektiven Untersuchungen und großen Querschnittsuntersuchungen an Kindern. Dabei werden Assoziationen zwischen entwicklungsneurotoxischen Effekten bei Kindern – wie Verminderung kognitiver Funktionen, Veränderung von Verhalten und Stimmung, Lerndefizite, Aufmerksamkeitsdefizite, Hyperaktivität, autistisches Verhalten, Verhaltensstörungen und Delinquenz – bei Blutbleikonzentrationen von unter 100 µg/l festgestellt. Mit zunehmender Blutbleikonzentration über 100 µg Blei/l Blut kommt es zusätzlich zu Veränderungen von neuromotorischen und neurosensorischen Funktionen sowie peripherer Neuropathie und Enzephalopathie. Es ist nicht möglich gewesen, eine NOAEC für die entwicklungsneurotoxische Wirkung von Blei abzuleiten (Greiner et al. 2022).

### 4.6 Genotoxizität

Es liegen keine Daten vor.

### 4.7 Kanzerogenität

#### 4.7.1 Fall-Kontroll-Studien

In einem Bleitetraethyl-produzierenden Betrieb (Du Pont) in Deepwater, New Jersey, USA (siehe Abschnitt 4.2), lag eine Exposition gegen Bleitetraethyl und anorganisches Blei vor. Bleitetraethyl wurde von 1923 bis 1991 produziert und ein Krebsregister wurde von 1956 bis 1987 geführt. Die Exposition gegen Bleitetraethyl wurde nach den verschiedenen Arbeitsbereichen in niedrig, mittel, hoch und sehr hoch eingeteilt und dann nach Expositionskonzentrationen und biologischem Monitoring (Urinmessung, Handschuhverwendung etc.) bemessen. Die kumulative Belastung wurde kalkuliert und die Beschäftigten in Quartile eingeteilt. Analysiert wurden die Daten zu den Tumoren in Gehirn, Niere, Lunge, Milz, Knochen und Verdauungssystem. Von 1062 Fällen mit der Diagnose Tumorerkrankung wurden 735 Personen in die Studie aufgenommen. Nicht aufgenommen wurden 270 Personen wegen fehlender Daten und 57 Frauen, da die Autoren deren Anzahl für nicht ausreichend erachteten. Bei den Tumoren im Verdauungssystem betrug das OR 1,3 (45 Fälle, 90-%-KI: 0,9–1,9) und in der Gruppe mit der höchsten kumulativen Bleitetraethyl-Exposition 2,2 (90-%-KI: 1,2–4,0). Bei den Beschäftigten mit Rektalkrebs, die jemals im Bereich mit Bleitetraethyl-Exposition gearbeitet haben, betrug das OR 3,7 (neun Fälle; 90-%-KI: 1,3–10,2), in der Gruppe mit der höchsten kumulativen Bleitetraethyl-Exposition 5,1 (sieben Fälle; 90-%-KI: 1,6–16,5) und nach 10-jähriger Latenzphase 4,9 (90-%-KI: 1,6–15,0). Bei Darmkrebs lag das OR bei 1,3 (16 Fälle; 90-%-KI: 0,7–2,5) und in der Gruppe mit der höchsten kumulativen Bleitetraethyl-Exposition bei 1,7 (acht Fälle; 90-%-KI: 0,8–4,0). Die Risiken für Darmkrebs waren damit nicht statistisch signifikant erhöht. Ebenso nicht bei Hodgkin-Lymphom mit einem OR von 2,7 (90-%-KI: 0,5–12,0). Die Kontrollen wurden in Bezug auf Geburtsjahr, Geschlecht, Gehaltsstufe und Diagnosejahr angepasst. Auch der Confounder Rauchstatus wurde berücksichtigt, soweit Daten verfügbar waren (31 % Fälle und 51 % Kontrollen) (Fayerweather et al. 1997; IARC 2006; Schwartz et al. 1993). Da bei der Bleitetraethyl-Produktion auch eine Exposition gegen anorganisches Blei vorlag, muss hier von einer Mischexposition ausgegangen werden.

#### 4.7.2 Kohortenstudien

Erhöhte Mortalität an Lungen-, Nieren- und Gehirntumoren sowie an Leukämie trat bei 1595 männlichen Arbeitern auf, die zwischen 1949 und 1982 in einer Raffinerie tätig waren. Inwieweit Bleitetraethyl an der Entstehung der Tumoren beteiligt ist, konnte aus den Daten nicht abgeleitet werden, da Mischexpositionen mit Gemischen von Kohlenwasserstoffen, Schwefelwasserstoff und weiteren Substanzen vorlagen (Bertazzi et al. 1989; Greim 1994).

Die Untersuchung der Mortalität an 2510 Beschäftigten einer Chemiefirma in Texas, USA, von 1952 bis 1977 ergab für die Jahre 1952–1960, in denen hauptsächlich Bleitetraethyl produziert wurde, ein standardisiertes Mortalitätsverhältnis (SMR) von 122 für Lungentumoren (13 Todesfälle; 95-%-KI: 73–194) und für Gehirntumoren von 186 (drei Todesfälle; 95-%-KI: 51–482) (IARC 2006).

## 5 Tierexperimentelle Befunde und In-vitro-Untersuchungen

### 5.1 Akute Toxizität

Die Daten zur akuten Toxizität beim Tier sind bei Henschler (1972 a) berichtet. Aufgeführt sind hier nur zusätzliche neu publizierte und bisher nicht erwähnte Studien.

#### 5.1.1 Inhalative Aufnahme

Nach einer einstündigen Exposition gegen 4,0–24,5 g Bleitetraethyl/m^3^ (3,1–15,7 g Pb/m^3^) in Toluol gelöst (50 % G/G) verendeten männliche Charles-River-Ratten (n = 2 pro Konzentration) ab einer Konzentration von 16,3 g/m^3^ nach zwei Tagen. In einem weiteren Versuch mit einstündigen Expositionen gegen 4,8; 7,8; 10,9 oder 16,3 g Bleitetraethyl/m^3^ (98,5 %) verendeten alle Tiere innerhalb von zwei Tagen, die gegen 10,9 und 16,3 g/m^3^ exponiert waren. Die Autopsie ergab pulmonale Ödeme und Kongestion bei den verendeten Tieren. Die letale Konzentration von Bleitetramethyl, in Toluol gelöst (50 % G/G), betrug unter gleichen Bedingungen 30,4 g/m^3^ (23,5 g Pb/m^3^) (Haskell Laboratories 1992 b).

#### 5.1.2 Orale Aufnahme

Nach intragastraler Verabreichung von 15–40 mg Bleitetraethyl/kg KG an je zehn nüchterne männliche Crl:CDO-Ratten wurde eine LD_50_ von 29 mg/kg KG ermittelt. Ab 20 mg/kg KG wurde Diarrhoe und ab 26 mg/kg KG Tremor beobachtet. Die Verabreichung von 17–28 mg/kg KG an nicht nüchterne männliche Crl:CDO-Ratten führte zu einer LD_50_ von 20 mg/kg KG. Bei den nicht nüchternen Ratten wurden bereits ab der niedrigsten Dosis Diarrhoe und Tremor beobachtet (Haskell Laboratories 1992 a).

Weibliche Ratten zeigten nach einmaliger intragastraler Gabe von 36 mg Trimethylblei/kg KG Tremor, Übererregbarkeit und Gewichtsverlust. Histopathologisch wurden Schäden im Hirnstamm, Neocortex und Hippocampus festgestellt (Greim 1995).

Schlundsondengaben von 3,4–130 mg Bleitetraethyl/kg KG (2,2–83 mg Pb/kg KG) in Erdnussöl an je eine männliche Ratte führten zu einer approximativen Letaldosis von 17 mg/kg KG (11 mg Pb/kg KG). Bei der höchsten subletalen Dosis von 12 mg/kg KG war das Tier ruhelos, schrie und verhielt sich unüblich. Die letale Dosis des Bleitetramethyls lag bei 108 mg/kg KG (83 mg Pb/kg KG). Subletale Konzentrationen bis 33 mg/kg KG (26 mg Pb/kg KG) führten zu Gewichtsverlust, Lethargie und einer leichten Diarrhoe (Haskell Laboratories 1992 b).

Männliche Fischer-Ratten (n = 8–10) erhielten Triethylbleichlorid per Schlundsonde. Die ermittelte LD_50_ betrug 14,5 mg/kg KG (Pryor et al. 1983). Die LD_50_ von **Bleiacetat** betrug nach oraler Gabe bei Ratten 4670 mg/kg KG (IFA 2023 a).

#### 5.1.3 Dermale Aufnahme

Nach Auftragen von 90–7500 mg Bleitetraethyl/kg KG in Ethylenbromid/Toluol auf die geschorene Haut von je einem männlichen Albino-Kaninchen verendeten alle Tiere ab einer Dosis von 1500 mg/kg KG (962 mg Pb/kg KG) innerhalb von 24 Stunden. Subletale Dosen führten zu Appetitlosigkeit und erhöhtem Speichelfluss. Bis 300 mg/kg KG traten in 14 Tagen Nachbeobachtung keine systemischen Effekte auf. Alle Tiere zeigten Reizungen an der behandelten Haut. Die letale Dosis betrug für Bleitetramethyl unter gleichen Bedingungen 6203 mg/kg KG (4808 mg Pb/kg KG) (Haskell Laboratories 1992 b).

#### 5.1.4 Subkutane Aufnahme

Nach einer subkutanen Injektion von 0, 3 oder 6 mg Triethylblei/kg KG (gelöst in Ethanol) an Nachkommen von zwölf Fischer-Ratten traten verminderte Körpergewichtsentwicklungen um 6 % und 13 % sowie frühe sensorische Defizite und Tremor auf. Bei den männlichen Tieren der hohen Dosisgruppe wurde Hypoaktivität und bei den behandelten weiblichen Tieren Veränderungen im affektiven Verhalten und Hyperaktivität beobachtet (Booze et al. 1983).

Eine Studie an neonatalen Ratten, denen am 5. Postnataltag Triethylbleichlorid subkutan in Dosierungen von 0; 4,5 oder 9 mg/kg KG (ca. 0; 2,8; 5,6 mg Pb/kg KG) injiziert wurde, zeigte, dass auch bei organischen Bleiverbindungen die neonatale Exposition zu ZNS-Schädigungen mit Hyperreaktivität und Schädigungen des Hippocampus (Entwicklungsstörungen) bei adulten Tieren führte. Die Effekte traten bereits in der niedrigen Dosisgruppe auf (Booze und Mactutus 1990; Hartwig 2009 a).

### 5.2 Subakute, subchronische und chronische Toxizität

#### 5.2.1 Inhalative Aufnahme

Je vier männliche Charles-River-Ratten wurden täglich eine Stunde an fünf aufeinanderfolgenden Tagen gegen 1,1 g Bleitetraethyl/m^3^ (0,7 g Pb/m^3^) oder 0,9 g Bleitetramethyl/m^3^ (0,7 g Pb/m^3^) exponiert. Ein Tier verendete drei Tage nach der letzten Exposition gegen Bleitetraethyl. Bei allen gegen Bleitetraethyl exponierten Tieren wurde Gewichtsverlust, Zittern, vermehrtes Rufen und Krampfen beobachtet. Die Bleitetramethyl-Exposition führte zu keinen Symptomen. Die Tiere wiesen erhöhte Bleigehalte in Knochen, Gehirn, Fettgewebe, Niere, Leber, Lunge und Milz auf. Die mikroskopische Untersuchung nach einer dreitägigen Nachbeobachtungsphase ergab, dass Bleitetramethyl stärkere Neuronen-Degenerationen und häufiger zerebrale Ödeme hervorrief als Bleitetraethyl. In der Milz, in der Leber und im Pankreas traten stärkere Effekte durch Bleitetraethyl auf. Bleitetraethyl verursachte zudem mehr Schädigungen in den Blutgefäßen und den Kapillaren der Lunge und eine stärkere Infiltration in die Bronchiolen als Bleitetramethyl. Das lymphoide Gewebe war leicht hypertroph durch Bleitetramethyl, während Atrophie bei den Bleitetraethyl-exponierten Tieren auftrat (k. w. A.; Haskell Laboratories 1992 b). Die mikroskopische Untersuchung ist nur knapp dargestellt. Die bei NTIS erhältliche Kopie des Studienberichts ist zum Teil nicht lesbar.

#### 5.2.2 Orale Aufnahme

Nach 28-tägiger Triethylbleichlorid-Gabe an fünf Tagen pro Woche per Schlundsonde an zehn männliche Fischer-Ratten betrug die LD_50_ 1,8 mg/kg KG und Tag. Zum Vergleich der Toxizitätsstärken erhielten Ratten **Bleiacetat**, das nach 28 Tagen (5 Tage/Woche) zu einer LD_50_ von 24,1 mg/kg KG und Tag führte. **Bleiacetat** wurde jedoch i.p. gegeben (Pryor et al. 1983).

Schlundsondengaben von 0,17 mg Bleitetraethyl/kg KG an fünf Tagen pro Woche führten bei Ratten nach 21 Wochen zu histologischen Veränderungen im ZNS und in der Leber (Henschler 1972 a).

Je fünf männliche Sprague-Dawley-Ratten erhielten 13 Wochen lang an fünf Tagen pro Woche per Schlundsonde 0; 0,2 oder 2 mg Bleitetraethyl/kg KG und Tag (in Wasser gelöst, 1 % Emulphor), 0; 0,2; 1,0 oder 2 mg Triethylbleiacetat/kg KG und Tag (nur in Wasser) oder 200 mg **Bleiacetat**/kg KG und Tag. Den Kontrolltieren wurde Wasser oder Vehikel verabreicht. Die Tiere der höchsten Dosisgruppen zeigten eine starke Abnahme des Körpergewichtes und wurden am 21. Tag (Triethylblei) und am 45. Tag (Bleitetraethyl) getötet. Weitere Untersuchungen der Tiere dieser Dosisgruppen wurden nicht durchgeführt. Bei den Tieren, die 0,2 mg Bleitetraethyl, 1,0 mg Triethylbleiacetat oder 200 mg **Bleiacetat**/kg KG und Tag erhielten, konnten ähnliche neuropathologische Läsionen im Gehirn und im Rückenmark (Wallersche Degenerationen) beobachtet werden. Im Lumbosakralnerv wurden Läsionen mit dosisabhängiger Steigerung ab 0,2 mg Triethylbleiacetat/kg KG und Tag beobachtet, die jedoch auch bei zwei Kontrolltieren in geringer Ausprägung auftraten. Die Autoren leiten daher einen NOEL für Triethylbleiacetat von 0,2 mg/kg KG und Tag ab. Statistisch signifikante hämatologische Effekte wurden ab 1,0 mg Triethylbleiacetat/kg KG und Tag und bei 200 mg **Bleiacetat**/kg KG und Tag gefunden. Die Blutentnahme erfolgte am 82. Tag (ECHA 2022 b; Franklin et al. 1987). Der LOAEL liegt bei 0,2 mg Bleitetraethyl/kg KG und Tag.

Je 20 junge männliche SD-Ratten (entwöhnt und zehn Tage akklimatisiert, vermutlich 31. Postnataltag, Gewicht 87 g) erhielten 13 Wochen lang an fünf Tagen pro Woche per Schlundsonde 0; 0,05; 0,1; 0,2; 0,5 oder 1,0 mg Triethylbleiacetat/kg KG und Tag oder 200 mg **Bleiacetat**/kg KG und Tag. Es wurden keine statistisch signifikanten Körpergewichtsentwicklungs-Veränderungen beobachtet, jedoch lag das Gewicht etwas niedriger bei 1 mg Triethylbleiacetat/kg KG und Tag und 200 mg **Bleiacetat**/kg KG und Tag. Die lichtmikroskopische Untersuchung von je fünf Tieren pro Dosisgruppe ergab Rückenmarksläsionen (Wallersche Degenerationen) im thorakalen und Lumbalraum ab 0,2 mg Triethylbleiacetat/kg KG und Tag, während mittels Transmissions-Elektronenmikroskopie bereits ab der niedrigsten Dosis von 0,05 mg/kg KG und Tag Läsionen nachweisbar waren. Die durch 200 mg **Bleiacetat**/kg KG und Tag oder 1 mg Triethylbleiacetat/kg KG und Tag hervorgerufenen Läsionen traten mit ähnlicher Häufigkeit und Schwere auf. Das relative Nierengewicht war in der höchsten Triethylbleiacetat-Dosisgruppe erhöht. Die histologische Untersuchung zeigte ab der niedrigsten Dosis Niereneffekte („Cyto eosin“, Pyknose) und im Serum erhöhte Phosphatwerte. Histologische Läsionen wurden in der höchsten Dosisgruppe der mit Triethylbleiacetat behandelten Tiere vermehrt in der Schilddrüse, der Leber, der Milz und dem Knochenmark beobachtet. Im Serum waren Cholesterin und die Aktivität der alkalischen Phosphatase ab 0,2 mg/kg KG und Tag erhöht. Ein Anstieg der Blutplättchen fand sich als einziger hämatologischer Effekt in der höchsten Dosisgruppe (Yagminas et al. 1990, 1992).

Männliche Fischer-Ratten (n = 9–10) erhielten Schlundsondengaben von 0; 0,35; 0,5; 0,73 oder 1,05 mg Triethylbleichlorid/kg KG und Tag in Sesamöl an fünf Tagen pro Woche über einen Zeitraum von 15 Wochen. Neurologische Verhaltenstests wurden in dreiwöchigen Abständen während der Verabreichung und nach drei und sechs Wochen in der Nachbeobachtungzeit ausgeführt. Nach sechswöchiger Gabe zeigte sich bei der thermalen Sensitivität ein signifikanter Effekt bei den Tieren der höchsten Dosisgruppe und nach zwölfwöchiger Gabe bereits ab der niedrigsten Dosis bei allen behandelten Tieren. Beim Test „multisensory conditioned avoidance response“ wurden signifikante Effekte nach Gabe der beiden höchsten Dosen beobachtet. Nach zum Vergleich der Toxizitätsstärke gegebenen intraperitonealen Dosen von 0; 2,3; 3,6; 5,2 oder 7,5 mg Bleiacetat/kg KG und Tag verendeten vier Tiere der höchsten Dosisgruppe nach 12–14 Wochen. Die Körpergewichtszunahme nach drei Wochen war bei allen behandelten Tieren ab 0,5 mg/kg KG statistisch signifikant erniedrigt. Eine statistisch signifikante Abnahme der motorischen Aktivität trat nach sechs Wochen in der höchsten Dosisgruppe auf. Die Autoren bewerten die Ergebnisse dahingehend, dass Triethylbleichlorid eine deutlich stärkere Neurotoxizität aufweist. Keine Unterschiede zu den Kontrolltieren ergaben sich bei den Untersuchungen der Griffstärke der Vorder- und Hinterbeine, des Gleichgewichtes, der Rektaltemperatur und dem Schreckverhalten (Pryor et al. 1983). Aufgrund der gewählten verschiedenen Aufnahmewege der organischen und anorganischen Bleiverbindungen ist ein quantitativer Vergleich ihrer Toxizität in seiner Aussagekraft begrenzt.

Eine sechsmonatige Bleitetraethyl- oder Bleitetramethyl-Schlundsondengabe von 0 oder 6 µg/kg KG, als Blei berechnet, an je zwei männliche und weibliche Rhesus-Affen (wild gefangen) pro Dosisgruppe führte zu keinen Symptomen (siehe Abschnitt 3.1; Heywood et al. 1979).

#### 5.2.3 Dermale Aufnahme

Hierzu liegen keine Daten vor.

#### 5.2.4 Intravenöse und subkutane Aufnahme

Eine siebentägige intravenöse Gabe von 6 mg **Bleitetraethy**l/kg KG und Tag in Ethanol an je zwei männliche und weibliche Rhesus-Affen (wild gefangen) führte zum Tod nach fünftägiger Gabe bei drei von vier Tieren. Diese Tiere zeigten gastrointestinale Störungen, reduzierte Reflexe, Muskelzittern sowie reduzierte Nahrungsaufnahme und eine Abnahme des Körpergewichtes. Die Aktivität der Cholinesterase im Gehirn betrug nur noch 62 %. Nach einer 14-tägigen intravenösen Gabe von 0,6–1,2 mg/kg KG und Tag an je zwei männliche und weibliche Rhesus-Affen traten auch nach einer 28-tägigen Nachbeobachtungszeit außer einem schwächeren Greifreflex keine Symptome auf. Von den mit 6 mg **Bleitetramethyl**/kg KG und Tag sieben Tage lang behandelten zwei männlichen und weiblichen Affen verendete kein Tier. Die Tiere waren nervös und übererregbar. Die histopathologische Untersuchung ergab axonale Degenerationen der peripheren Nerven. Die zweiwöchige intravenöse Gabe von 1,2–2,4 mg Bleitetramethyl/kg KG und Tag führte nach vier Tagen zu einem reduzierten Patellar-Reflex, diese Einschränkung trat nach einer 28-tägigen Nachbeobachtungszeit nicht mehr auf (Heywood et al. 1978). Bleitetraethyl zeigte eine stärkere toxische Wirkung als Bleitetramethyl.

### 5.3 Wirkung auf Haut und Schleimhäute

Nach Auftragen von 90–2250 mg Bleitetraethyl/kg KG in Ethylenbromid/Toluol auf die geschorene Haut von Albino-Kaninchen hatten alle Tiere Reizungen an der behandelten Haut. Reizwirkungen durch das Ethylenbromid traten bis 2250 mg/kg KG nicht auf (Haskell Laboratories 1992 b). Toluol verursachte an der Haut nur eine mäßige Reizwirkung (Henschler 1986).

### 5.4 Allergene Wirkung

Es liegen keine Daten vor.

### 5.5 Reproduktionstoxizität

#### 5.5.1 Fertilität

Generationenstudien und Fertilitätsstudien sind nicht durchgeführt worden.

Im REACH-Registrierungs-Dossier wird ein Dominant-Letal-Test berichtet. Zehn männliche Mäuse (k. A. zum Stamm) pro Gruppe erhielten jeweils eine einzelne Dosis Bleitetraethyl oral (6,48 und 32 mg/kg KG) oder intraperitoneal (0,65 und 3,2 mg/kg KG) und wurden anschließend mit vier weiblichen unbehandelten Tieren pro Woche sechs Wochen lang verpaart. Der Präimplantationsverlust war nach der intraperitonealen Gabe bei 3,2 mg/kg KG zwischen der 3. und 6. Verpaarungswoche erhöht. Eine signifikante Zunahme früher Resorptionen wurde nicht beobachtet. Die orale Gabe von 32 mg/kg KG hatte während der ersten beiden Wochen einen reduzierten Verpaarungsindex zur Folge (ECHA 2022 b). Aufgrund der limitierten Angaben kann die Studie nicht zur Bewertung herangezogen werden.

#### 5.5.2 Entwicklungstoxizität

Neue Studien sind seit Erscheinen der Nachträge zu Bleitetraethyl und Bleitetramethyl (Hartwig 2009 a, b) nicht veröffentlicht worden. Die bewertungsrelevanten Studien werden hier kurz dargestellt.

##### 5.5.2.1 Pränatale Entwicklungstoxizität

In Studien zur pränatalen Entwicklungstoxizität führte Bleitetraethyl bei Ratten (6. bis 16. Gestationstag) und Mäusen (5. bis 15. Gestationstag) nach Schlundsondengabe ab 1 mg/kg KG und Tag (0,64 mg Pb/kg KG und Tag) zu einer erhöhten Zahl an Resorptionen und einer erniedrigten Zahl lebender Feten sowie bei Mäusen zusätzlich zu einer verzögerten Skelettentwicklung. Ab dieser Dosis war die maternale Körpergewichtszunahme erniedrigt. Der NOAEL für Entwicklungs- und Maternaltoxizität lag für beide Spezies bei 0,1 mg/kg KG und Tag (0,064 mg Pb/kg KG und Tag). Bis 10 mg/kg KG und Tag wurden bei beiden Spezies keine teratogenen Effekte beobachtet (Hartwig 2009 a; Kennedy et al. 1975).

Zu Bleitetramethyl liegt keine Studie zur pränatalen Entwicklungstoxizität nach gültigen Prüfrichtlinien vor.

##### 5.5.2.2 Entwicklungsneurotoxizität

Für Bleitetraethyl und Bleitetramethyl sind keine aussagekräftigen Studien zur Entwicklungsneurotoxizität durchgeführt worden. Die einmalige subkutane Gabe von Triethylblei, dem Metaboliten von Bleitetraethyl, am 5. Postnataltag führte bei Ratten ab der niedrigsten getesteten Dosis von 4,5 mg Triethylbleichlorid/kg KG (ca. 2,8 mg Pb/kg KG) zu ZNS-Schädigungen mit Hyperreaktivität und Schädigungen des Hippocampus bei adulten Tieren (Booze und Mactutus 1990; Hartwig 2009 a).

Im BAT-Addendum von 2022 zu **Blei und seinen anorganischen Verbindungen** ist umfassend die bleiinduzierte Entwicklungstoxizität und Entwicklungsneurotoxizität bei Tieren beschrieben. So kommt es neben Effekten auf das Skelett zu morphologischen und funktionellen Effekten auf das periphere und zentrale Nervensystem (Greiner et al. 2022).

### 5.6 Genotoxizität

#### 5.6.1 In vitro

Bleitetraethyl ist nicht mutagen im bakteriellen Mutagenitätstest, jedoch zeigten sich signifikant erhöhte Schwesterchromatidaustauschraten (SCEs) sowie ein Einfluss auf die Funktion des Spindelapparates ab einer Konzentration von 0,29 mg Triethylbleichlorid/l in humanen Lymphozyten (Grandjean und Andersen 1982; Greim 1994).

Bleitetramethyl ist ebenfalls nicht mutagen in bakteriellen Mutagenitätstests (Greim 1995).

Das Auftreten einer Spindelstörung nach Inkubation von V79-Zellen mit „extract of particulate matter“ (EPM) aus Benzinmotor-Auspuffabgasen wird auf die in EPM enthaltenden organischen Bleiverbindungen zurückgeführt (Hadnagy und Seemayer 1988).

Mit Triethylbleiacetat inkubierte CHO-Zellen zeigten Zytotoxizität bereits ab 10 µmol/l ohne bzw. ab 80 µmol/l mit Zugabe eines metabolischen Aktivierungssystems. Chromosomenaberrationen traten ohne Zugabe eines metabolischen Aktivierungssystems ab 6 µmol Triethylbleiacetat/l und mit Aktivierung ab 40 µmol Triethylbleiacetat/l auf. Die Autoren bewerten die Trialkylblei-Verbindung als ein potentes Klastogen und diskutieren, dass die Abnahme der zytotoxischen und klastogenen Wirkung in Anwesenheit eines metabolischen Aktivierungssystems möglicherweise auf der Bindung der Testsubstanz an Komponenten des Aktivierungsgemisches beruht statt auf einer Metabolisierung zu einer weniger toxischen bzw. weniger klastogen wirkenden Substanz (Blakey et al. 1992).

#### 5.6.2 In vivo

Im Dominant-Letaltest mit männlichen Mäusen war Bleitetraethyl nach oraler oder i.p. Gabe negativ. Nach Gabe an Larven von Drosophila melanogaster und anschließender Paarung traten klastogene Effekte (X-Chromosomenverlust) sowie eine Spindelstörung bei höheren Konzentrationen auf (XXY-Individuen). Auch für Triethylblei ist eine Wirkung auf den Spindel-Mechanismus beschrieben, der zu einer Fehlverteilung der Chromosomen in den Tochterzellen führte (Greim 1994).

### 5.7 Kanzerogenität

#### 5.7.1 Kurzzeitstudien

Hierzu liegen keine Daten vor.

#### 5.7.2 Langzeitstudien

Nach subkutaner Injektion (post partum) von insgesamt 0,6 mg Bleitetraethyl an 41 weibliche Mäuse, entwickelten sich bei fünf Tieren (12 %) nach 36 Wochen maligne Lymphome (Kontrolle: 0 von 48). Bei den männlichen Tieren dieser Dosisgruppe entwickelten sich bei einem von 26 Tieren Lymphome (Kontrolle: 1 von 46). Während hinsichtlich anderer Tumoren keine signifikanten Unterschiede zwischen Kontroll- und Dosisgruppe gesehen wurden, ist die Tumorinzidenz für die malignen Lymphome bei weiblichen Tieren signifikant (p < 0,05) erhöht (Epstein und Mantel 1968; Greim 1994). Die Studie ist wegen unzureichender Dokumentation nicht zur Bewertung von Bleitetraethyl heranziehbar.

### 5.8 Sonstige Wirkungen

Mit dem Trinkwasser, das Bleitetraethyl in Konzentrationen von 0; 0,5; 1,0; 2,0 oder 10 mg/l (als Blei berechnet, mit Tween 80 als Emulgator) enthielt, erhielten je zehn männliche Swiss-Cross-Mäuse drei Wochen lang eine tägliche Bleitetraethyl-Dosis von ca. 0; 0,1; 0,2; 0,4 oder 2,0 mg/kg KG (als Blei berechnet). Bereits bei der niedrigsten Dosis traten immunsuppressive Effekte auf (Blakley et al. 1980).

Nach 14-tägiger Schlundsondengabe von Bleitetraethyl wurde eine Hypersensibilisierung vom verzögerten Typ sowie eine erhöhte Leukozytenanzahl beobachtet (k. w. A.; Luster et al. 1992).

## 6 Bewertung

Kritischer Effekt ist die starke Neurotoxizität beim Menschen.

Es liegen keine neuen Humanstudien mit ausschließlicher Exposition gegen Bleitetraethyl, Bleitetramethyl oder eine andere organische Bleiverbindung vor. Seit dem Jahr 2000 ist das Inverkehrbringen von verbleitem Ottokraftstoff gesetzlich verboten. Damit sind keine neuen Studien zu Bleitetraalkylen zu erwarten.

**MAK-Wert. **Urin- und Faecesanalysen nach Exposition gegen Bleitetraethyl oder Bleitetramethyl zeigen eine Metabolisierung zu anorganischem Blei. Es ist davon auszugehen, dass die so entstandenen anorganischen Blei-Ionen im Körper die gleichen Wirkungen verursachen, die nach direkter Aufnahme von anorganischen Bleiverbindungen beobachtet werden.

Organische Bleiverbindungen rufen nach oraler Aufnahme oder bei Inkubation isolierter Gewebe stärkere neurotoxische Effekte hervor als Blei-Ionen (siehe Abschnitt 2 und 5.2.2). Die deutlich lipophileren Eigenschaften von Bleitetraethyl und Bleitetramethyl führen im Vergleich zu Blei-Ionen zu einer höheren Aufnahme ins Gehirn und lassen eine bessere Aufnahme in Neuronen vermuten, die die stärkeren neurotoxischen Effekte erklären kann.

Die inhalative Retention von Bleitetraethyl bzw. Bleitetramethyl ist mit 30 % ähnlich wie die von anorganischen Bleiverbindungen (siehe Abschnitt 3.1).

In der im Nachtrag 1994 zur Ableitung eines Grenzwertes herangezogenen Studie von Zhang et al. (1994) lag in der Gruppe der gegen Bleitetraethyl-Konzentrationen von 0,03–0,049 mg/m^3^ (als Blei berechnet) Exponierten (Mittelwert 0,04 mg/m^3^) die Fallzahl mit Tremor nicht höher als in der Kontrollgruppe. Da jedoch Tremor einen bereits schwerwiegenden Effekt darstellt, sensitivere Endpunkte nicht gemessen wurden und die Gruppe der niedrig Exponierten nur 27 Personen umfasste, wird die Studie nur zur Unterstützung eines Grenzwertes herangezogen. Die Studie zeigt jedoch, dass bei Bleitetraethyl und Bleitetramethyl gravierende akute Effekte bei Luftkonzentrationen unterhalb von 0,05 mg/m^3^ weitgehend ausgeschlossen werden können.

Die Tierdaten sind nicht für eine Ableitung eines Grenzwertes geeignet, da auch bei der niedrigsten eingesetzten Dosis von 0,05 mg Triethylbleiacetat/kg KG und Tag (13-Wochen-Studie) noch schwerwiegende Effekte beobachtet wurden.

Bei Blei und seinen anorganischen Verbindungen war der empfindlichste Endpunkt ebenfalls die Neurotoxizität, wie in Humanstudien verlässlich nachgewiesen wurde. Es konnte aus dem BAT-Wert von 150 µg Pb/l Blut mithilfe eines PBPK-Modells ein MAK-Wert von 0,004 mg Blei/m^3^ abgeleitet werden (Greiner et al. 2022; Hartwig und MAK Commission 2022).

Da verlässliche Daten zu Bleitetraethyl, Bleitetramethyl und weiteren organischen Bleiverbindungen fehlen, die eine Grenzwert-Ableitung erlauben würden, muss mindestens der MAK-Wert für Blei und seine anorganischen Verbindungen eingehalten werden. Daher wird dieser Wert übernommen und der MAK-Wert von Bleitetraethyl und Bleitetramethyl auf 0,004 mg/m^3^, als Blei berechnet, abgesenkt. Dieser MAK-Wert gilt auch für weitere organische Bleiverbindungen.

Zur Abbildung der Bleibelastung durch organische und anorganische Bleiverbindungen dient die Messung der Blutbleikonzentration. Der BAT-Wert von 150 µg Blei/l Blut muss unbedingt eingehalten werden (Greiner et al. 2022).

**Spitzenbegrenzung. **Der kritische Effekt der organischen Bleiverbindungen ist die starke neurotoxische Wirkung. Die Zuordnung zu Spitzenbegrenzungs-Kategorie II wird beibehalten. Die im Vergleich mit den anorganischen Bleiverbindungen stärkere neurotoxische Wirkung der organischen Bleiverbindungen findet in der deutlich höheren Lipophilie und der dadurch bedingten leichteren Aufnahme in das Gehirn eine plausible Erklärung. Die Aufenthaltszeit („mean residence time“) der organischen Bleiverbindungen im Blut beträgt 13 Sekunden (Heard et al. 1979). Aufgrund der kurzen Halbwertszeit der organischen Bleiverbindungen im Blut und ihrer im Vergleich zu den anorganischen Verbindungen leichteren Aufnahme in das Gehirn wird ein Überschreitungsfaktor von 1 festgelegt.

**Fruchtschädigende Wirkung. **Für organische Bleiverbindungen liegen zur Entwicklungstoxizität und Entwicklungsneurotoxizität keine belastbaren Daten beim Menschen vor.

In Studien zur pränatalen Entwicklungstoxizität führt Bleitetraethyl bei Ratten und Mäusen nach intragastraler Gabe ab 1 mg/kg KG und Tag (0,64 mg Pb/kg KG und Tag) zu einer erhöhten Zahl an Resorptionen und einer erniedrigten Zahl lebender Feten sowie bei Mäusen zusätzlich zu einer verzögerten Skelettentwicklung. Der NOAEL für Entwicklungstoxizität liegt für beide Spezies bei 0,1 mg/kg KG und Tag (0,064 mg Pb/kg KG und Tag). Bis 10 mg/kg KG und Tag sind bei beiden Spezies keine teratogenen Effekte zu beobachten gewesen (Kennedy et al. 1975). Zu Bleitetramethyl liegt keine Studie zur pränatalen Entwicklungstoxizität nach gültigen Prüfrichtlinien vor. 

Für organische Bleiverbindungen sind keine validen Studien zur Entwicklungsneurotoxizität durchgeführt worden.

In vivo wirken organische Bleiverbindungen stärker neurotoxisch als anorganische Bleiverbindungen (Abschnitt 2 und 5.2.2).

Im BAT-Addendum von 2022 ist ausführlich dargelegt, dass ausreichend konsistente Evidenz aus prospektiven Untersuchungen und großen Querschnittsuntersuchungen an Kindern zur entwicklungstoxischen und entwicklungsneurotoxischen Wirkung von Blei vorhanden ist. Es ist nicht möglich gewesen, einen NOAEL für Entwicklungsneurotoxizität abzuleiten (Greiner et al. 2022). Blei und seine anorganischen Verbindungen sind bei einem MAK-Wert von 0,004 mg Blei/m^3^ bzw. bei einem BAT-Wert von 150 µg Blei/l Blut der Schwangerschaftsgruppe A zugeordnet worden (Greiner et al. 2022; Hartwig und MAK Commission 2022).

Für organische Bleiverbindungen wird aufgrund der Metabolisierung und Anreicherung von Blei in Analogie zu Blei und seinen anorganischen Verbindungen bei einem MAK-Wert von 0,004 mg/m^3^ eine Zuordnung zu Schwangerschaftsgruppe A vorgenommen.

**Krebserzeugende Wirkung. **Es liegen keine Studien vor, die eine humankanzerogene Wirkung klar belegen. Ebenso fehlen Zwei-Jahre-Kanzerogenitätsstudien an Tieren (Nagern). Eine klastogene Wirkung von Bleitetraethyl wurde in vitro beobachtet und eine aneugene Wirkung ist bei Drosophila belegt.

Da gezeigt wurde, dass beim metabolischen Abbau von Bleitetraethyl und Bleitetramethyl anorganisches Blei entsteht, werden organische Bleiverbindungen in Analogie zu Blei und seinen anorganischen Verbindungen in die Kanzerogenitäts-Kategorie 4 eingestuft.

**Keimzellmutagene Wirkung. **Es liegen keine bewertungsrelevanten Daten zur Einstufung in eine Kategorie für Keimzellmutagene für organische Bleiverbindungen vor.

Organische Bleiverbindungen werden in Analogie zu Blei und seinen anorganischen Verbindungen in die Kategorie 3 A eingestuft.

**Hautresorption. **Bleialkyle werden zu 6 % über die Haut aufgenommen (Greim 1994). Daten zur Toxizität nach chronischer dermaler Exposition der Verbindungen liegen nicht vor. Nach Modellberechnungen werden 3,44 mg bzw. 79,3 mg Bleitetramethyl (entspricht 2,67 mg bzw. 61,5 mg Blei) sowie 0,01 mg bzw. 0,3 mg Bleitetraethyl (entspricht 0,0064 mg bzw. 0,19 mg Blei) über die Haut aufgenommen. Beim MAK-Wert von 0,004 mg Blei/m^3^ würden in acht Stunden etwa 0,016 mg Blei aufgenommen bzw. 0,012 mg retiniert. Da für beide Stoffe mindestens ein Modell dermale Penetrationsmengen berechnet, die in der gleichen Größenordnung wie die inhalative Aufnahme unter MAK-Wert-Bedingungen liegen oder diese sogar überschreiten, wird die „H“-Markierung für organische Bleiverbindungen beibehalten.

**Sensibilisierende Wirkung. **Zur sensibilisierenden Wirkung von organischen Bleiverbindungen liegen weiterhin keine Befunde beim Menschen und keine Ergebnisse aus tierexperimentellen Untersuchungen oder aus Untersuchungen mit alternativen Testverfahren (NAMs) vor. 

Organische Bleiverbindungen werden daher weiterhin weder mit „Sh“ noch mit „Sa“ markiert.
